# A novel *Schinkia* sp. respires nitrous oxide over a broad pH range

**DOI:** 10.1128/aem.00069-26

**Published:** 2026-05-26

**Authors:** Guang He, Megan E. Davin, Shaylan Kolodney, Emily Stutzman, Asher Wright, Robert L. Hettich, Frank E. Löffler

**Affiliations:** 1 Department of Civil and Environmental Engineering, University of Tennessee 4292, Knoxville, Tennessee, USA; 2 Department of Biosystems Engineering and Soil Science, University of Tennessee 4292, Knoxville, Tennessee, USA; 3 Bredesen Center for Interdisciplinary Research and Graduate Education, University of Tennessee 4292, Knoxville, Tennessee, USA; 4 Biosciences Division, Oak Ridge National Laboratory 6146, Oak Ridge, Tennessee, USA; 5 Caney Fork Farms, Carthage, Tennessee, USA; 6 Department of Biochemistry & Cellular and Molecular Biology, University of Tennessee 847214 , Knoxville, Tennessee, USA; Georgia Institute of Technology, Atlanta, Georgia, USA

**Keywords:** NosZ, N_2_O reduction, acidic pH, alkaline pH, denitrification, greenhouse gas, *Schinkia*, *Calidifontibacillus*, soil microbiology

## Abstract

Known bacteria reduce the climate-active gas nitrous oxide (N_2_O) to dinitrogen (N_2_) over narrow pH ranges, limiting their efficacy in pH heterogeneous soil environments. A dual pH (pH 4.5 → 7.0 → 4.5) enrichment strategy with N_2_O as electron acceptor yielded a novel *Schinkia* isolate, strain CFF1, capable of N_2_O reduction over broad pH (4.5–8.5) and salinity (1–20 g L^−1^ NaCl) ranges. Strain CFF1 couples complete fumarate oxidation with N_2_O respiration and produces 10.2 ± 2.4 mg of biomass (dry weight) per mmol N_2_O consumed. Fumarate, succinate, lactate, pyruvate, and propionate, but not acetate, formate, or hydrogen, support N_2_O respiration. N_2_O reduction rates of 6.13 ± 0.30 µmol N_2_O (mg biomass hour)^−1^ were measured at pH 7.5, with ~30% lower rates observed at pH 4.5 and 8.5. Acetylene (33%, vol/vol), a potent inhibitor of N_2_O reductase (NosZ), had no impact on the growth of strain CFF1 with N_2_O. Alternate growth-supporting electron acceptors include nitrate, nitrite, and O_2_. Genomic investigations revealed four distinct clade II *nosZ* genes, and proteomic measurements demonstrated the synthesis of the same two NosZ under both acidic and alkaline growth conditions. Physiological characterization and phylogenomic analysis indicate the isolate represents a new species in the genus *Schinkia*, for which the name *Schinkia acidoalkalinis* strain CFF1 is proposed. A soil isolate capable of reducing N_2_O over a broad pH range expands our understanding of the ecophysiology of N_2_O-reducing bacteria and N_2_O turnover in environments where pH varies across space and time.

**IMPORTANCE:**

Dissimilatory N_2_O reduction catalyzed by the N_2_O reductase NosZ is the major process consuming the climate-active gas N_2_O. Known N_2_O-reducing microorganisms operate within narrow pH windows, potentially limiting N_2_O turnover in heterogeneous soil environments. We isolated *Schinkia acidoalkalinis* strain CFF1 capable of reducing N_2_O at similar rates over a broad pH range spanning pH 4.5–8.5. The discovery of an N_2_O-reducing pH generalist expands opportunities for developing robust biotechnologies to enhance N_2_O consumption in natural and engineered systems characterized by pH heterogeneity.

## INTRODUCTION

Nitrous oxide (N_2_O, laughing gas) is a potent climate-active gas with a warming potential nearly 300 times greater than that of carbon dioxide (CO_2_) over a 100 year time span (1). Anthropogenic activities have led to a 40% increase in global N_2_O emissions over the past four decades, and the atmospheric N_2_O concentration currently increases by 0.4% per year, making N_2_O the fastest growing greenhouse gas (2). Beyond its role as a climate-active gas, a growing body of research highlights that N_2_O can impact microbial processes, such as methionine biosynthesis, methanogenesis, organohalide respiration, sulfate reduction, and methylmercury formation, by inhibiting the activity of cobamide-dependent enzymes (3–7). Thus, N_2_O emissions influence not only climate forcing but also microbial community function and ecosystem-level biogeochemical transformations. The major cause of rising N_2_O emissions is the intensive use of synthetic nitrogen (N) fertilizers for the ever-increasing global demand for feed and food (8). The overuse of synthetic fertilizer provides a surplus of fixed N (i.e., nitrate [NO_3_^−^], ammonium [NH_4_^+^]) and these N pools fuel microbial and abiotic processes that generate N_2_O, accelerating emissions to the atmosphere (9).

Microbial processes, foremost denitrification, nitrification, coupled biotic-abiotic chemodenitrification, and abiotic photochemodenitrification are major biogeochemical processes generating N_2_O from fixed N (10, 11). N_2_O consumption is restricted to microorganisms expressing N_2_O reductase (NosZ), with a minor contribution from direct N_2_O assimilation (12–14). The sustained increase in atmospheric N_2_O suggests that ecosystem-scale N_2_O turnover is not balanced, presumably reflecting the stimulation of N_2_O production by surplus fixed N and environmental constraints on N_2_O-consuming microbiomes. Traditionally, microbial N_2_O reduction has been considered the terminal step of denitrification, but it has become evident that a diversity of N_2_O reducers are non-denitrifiers (14, 15). The rates of N_2_O formation and consumption are influenced by an interplay of biogeochemical factors, including redox potential, pH, electron donor availability, and supply of copper ions to enable NosZ activity (5, 16). Laboratory experiments demonstrated that acidic pH can lead to higher net N_2_O emissions by disrupting the synthesis (maturation) of functional NosZ (17) or delaying the initiation of N_2_O reduction (18). These intricacies highlight the need for an improved understanding of N_2_O-reducing microbiomes, potentially yielding robust approaches to mitigate N_2_O emissions from critical ecosystems, such as agricultural soils, wastewater treatment plants, and tropical forest soils.

Soil microbiome engineering by inoculation of microorganisms with desired function(s) (i.e., bioaugmentation) is an emerging field (19), with great potential for enhancing crop growth (20) and reducing nitrogen loss (e.g., in the form of N_2_O emissions) (21, 22). Although promising, bioaugmentation faces several challenges, including the ability of inoculated microorganisms to compete and establish themselves within the native soil microbiome (23). A key issue challenging N_2_O mitigation strategies is the narrow pH window of characterized N_2_O-reducing bacteria, and well-studied soil isolates do not show sustained N_2_O reduction activity below pH 6.0 (Table 1) (17, 24–26). Notably, recent studies have reported that some soil bacteria reduce N_2_O under acidic conditions (Table 1), although these organisms show no activity at circumneutral and alkaline pH (27–29). These observations suggest that the N_2_O reducers grow within narrow pH windows, complicating efforts to control emissions across habitats (e.g., soils) that experience variable and heterogeneous pH conditions. Given the inherent heterogeneity of soils and pH dynamics (30), bacteria that effectively reduce N_2_O over a broader pH range are desirable. Such pH generalists represent a resilient N_2_O sink under fluctuating pH conditions and could enable robust biotechnological solutions for mitigating N_2_O emissions from (agricultural) soils and other natural and engineered ecosystems.

**TABLE 1:** pH ranges reported for N_2_O reduction by representative N_2_O-reducing bacteria

Organism^*a*^	pH range	*nosZ* type	Reference
*Thioalkalivibrio denitrificans* strain ALJD	7.5–10.5	I	(31)
*Alicycliphilus denitrificans* strain I51	6–9	I	(32)
*Shewanella loihica* strain PV-4	6–8	I	(26)
*Bradyrhizobium diazoefficiens* strain 110*spc*4	6–8	I	(33)
*Ensifer meliloti* strain 1021	7–8	I	(25)
*Paracoccus denitrificans* strain DSM 314	6.2–7.5	I	(17)
*Bacillus* spp. (multiple isolates)	6–7	II	(24)
*Cloacibacterium* sp. strain CB-01	6.5–7	II	(34)
***Paraburkholderia* spp. (multiple isolates**)	**4.8–6.8**	**I**	(29)
***Trinickia* sp. strain Z7**	**4.2–7.1**	**I**	(29)
***Nitratiruptor labii* strain HRV44**	**5.4–6.4**	**II**	(35)
***Rhodanobacter* sp. strain C01**	**5.7**	**I**	(36)
***Desulfosporosinus nitrosoreducens* strain PR**	**4.5–6**	**II**	(27)
***Methylocella tundrae* strain T4**	**5.5**	**I**	(28)
***Methylacidiphilum caldifontis* strain IT6**	**2.0**	**II**	(28)
***Desulfitobacterium nosdiversum* strain Sab5**	**4.5–6.5**	**III**	(14)
*Sporomusa acidovorans* strain Mol	6.5–7.5	III	(14)

^*a*^ The bacteria listed represent axenic cultures, except *Desulfosporosinus nitrosoreducens*, a strict anaerobe characterized in a co-culture (27); *Desulfitobacterium nosdiversum* was characterized in an enrichment culture (14). Organisms highlighted in bold can reduce N_2_O under acidic conditions.

**Fig 1:**
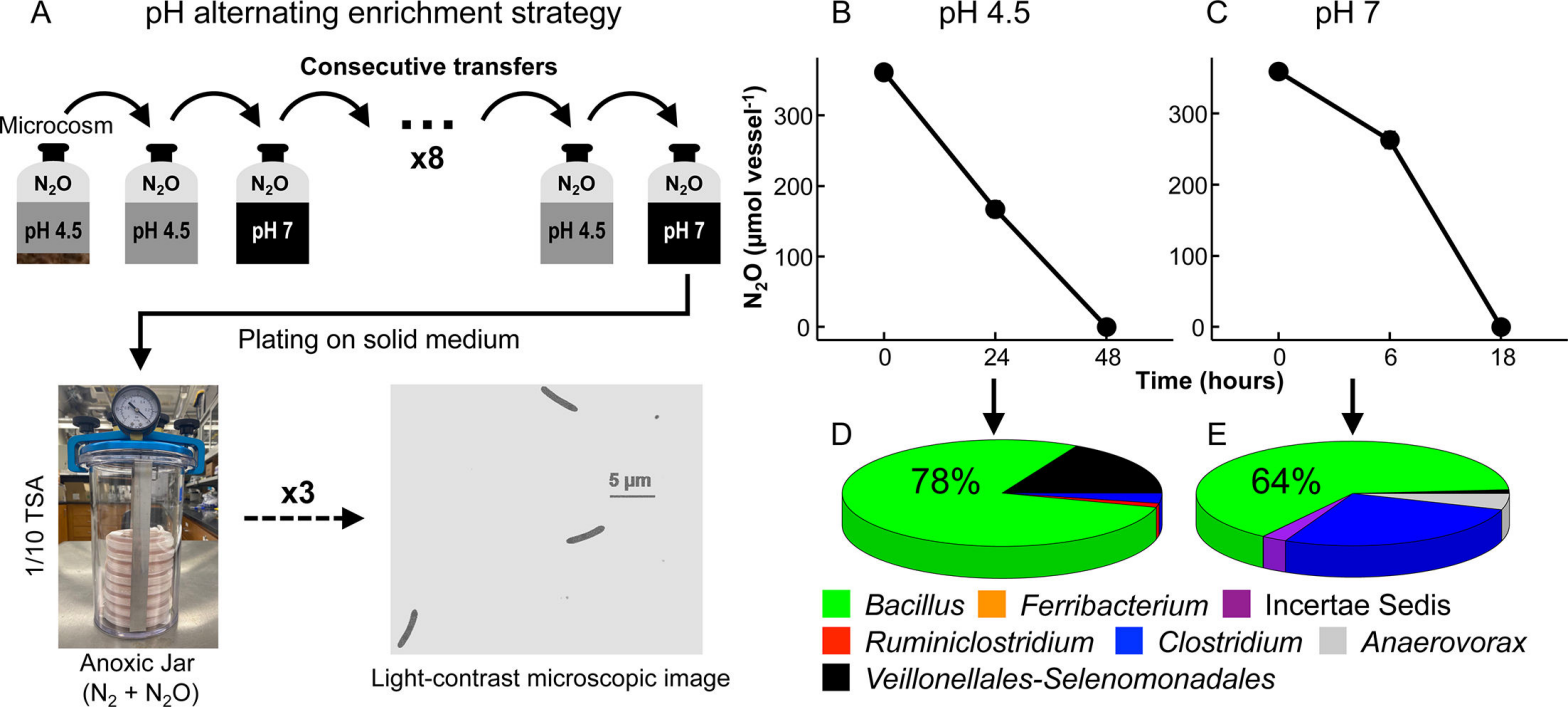
Dual-pH enrichment strategy yields an N_2_O-reducing bacterial soil isolate, strain CFF1. Panel **A **illustrates the enrichment and isolation procedure, starting with an N_2_O-reducing CFF soil microcosm, and subsequent transfers (12 total) in defined basal salt medium with alternating pH 4.5 and pH 7 growth conditions. Panels **B** and **C **show N_2_O reduction performance of 6th-generation transfer cultures in pH 4.5 (left) and pH 7 (right) medium. The data represent averages, and the standard deviations depict performance variations of triplicate cultures. Error bars are not visible because they are smaller than the symbols. The pie charts show 16S rRNA gene amplicon sequence data illustrating the microbial community composition and the relative taxonomic abundances in 6th generation transfer cultures grown at pH 4.5 (panel **D**) and at pH 7 (panel **E**).

In this study, we adopted a dual enrichment strategy (34) aimed at selecting N_2_O-reducing pH generalists. We hypothesized that by alternating acidic (pH 4.5) and neutral (pH 7) conditions, the enrichment process will select for microorganisms capable of reducing N_2_O over a broad pH range (Fig. 1A). This approach was applied to microbial communities derived from soil (pH ~7) collected at Caney Fork Farms (CFF), a farm practicing regenerative agriculture, with the goal of cultivating and identifying N_2_O-respiring pH generalists.

## MATERIALS AND METHODS

### Soil sampling and microcosms

Soil samples were collected from rotationally grazed chestnut orchards, a form of silvopasture, at CFF (https://www.caneyforkfarms.com/) in Carthage, Tennessee (TN) (37). CFF implements no-till regenerative farming, does not use synthetic fertilizers, and has multiyear soil and tree data. Soil samples were collected from 5 to 20 cm depth in October 2020, transferred to individual, sterile plastic bags, placed inside a cooler, and transported to the laboratory the same day. Soil pH (~6.96) was measured following mixing of 2.5 g of wet soil with 10 mL deionized water. Soil microcosms were established within 3 days following soil collection in 160 mL glass serum bottles containing 100 mL of anoxic, completely synthetic, defined basal salt medium (14) (Methods S1). Preliminary experiments tested different potential electron donors, including acetate, formate, propionate, ethanol, methanol, and fumarate (5 mM each). Robust N_2_O reduction activity was observed in fumarate-amended microcosms, and subsequent cultivation efforts used 5 mM fumarate (unless noted otherwise), which was added from an autoclaved 1 M stock solution, as electron donor and carbon source. Ten milliliters of N_2_O gas (416 µmol, 4.16 mM nominal; 99.5%) was added using a 10 mL syringe equipped with a 0.2 µm polyethersulfone membrane filter (Thermo Fisher Scientific, Waltham, MA, USA). Following a 2-hour equilibration period, periodic N_2_O measurements started. N_2_O reduction commenced following a 1-week lag phase, and N_2_O was replenished when consumed. A soil microcosm that had consumed three repeated N_2_O feedings (~1,200 µmol total) was selected for further enrichment efforts.

### Enrichment in medium with alternating pH

The enrichment procedure was initiated by transferring 3% (vol/vol) microcosm suspension to fresh pH 4.5 medium. Following complete consumption of two repeated N_2_O feedings, the pH 4.5 enrichment culture was transferred to pH 7 medium (3%, vol/vol). This pH-alternating transfer procedure was repeated six times (six transfers at pH 4.5, six transfers at pH 7, Fig. 1A). The pH 7 medium was prepared as described for the pH 4.5 medium, but a lower amount of KH_2_PO_4_ (0.2 g L^−1^) was used, and the medium was buffered with bicarbonate (2.6 g L^−1^, 30 mM). The final medium pH was adjusted to ~7 with CO_2_.

### Community analysis of pH 4.5 and pH 7.0 enrichment cultures

16S rRNA gene amplicon sequencing was performed on samples collected from 6th generation transfer cultures grown at pH 4.5 and at pH 7, following complete N_2_O consumption. Cells from 1 mL of culture suspension samples were collected by centrifugation (10,000 × *g*, 20 min, 4°C), and genomic DNA was extracted from the pellets using the DNeasy PowerSoil Kit (Qiagen, Hilden, Germany). 16S rRNA gene-based amplicon sequencing was conducted at the University of Tennessee (UT) Genomics Core following established procedures (38). Amplification used the primer set (341F, CCTACGGGNGGCWGCAG; 785R, GACTACHVGGGTATCTAATCC) targeting the V3–V4 hypervariable regions of the bacterial 16S rRNA gene. Analysis of amplicon reads was conducted using nf-core/ampliseq v2.3.1 with Nextflow (Methods S2) (39).

### Isolation and cultivation of strain CFF1

Efforts to obtain N_2_O-reducing isolates potentially capable of growth with N_2_O over a broader pH range were conducted using 1/10 strength tryptic soy agar (TSA) plates. A 1 mL sample of a 6th-generation pH 4.5 transfer culture that actively reduced N_2_O was serially diluted 10-fold in basal salt medium, and 100 µL of cell suspension aliquots were evenly spread on the TSA plates. The plates were incubated under an atmosphere of N_2_/N_2_O (9/1, vol/vol). Colony formation was observed after 24 h, and after a 48-hour incubation period, single colonies were streaked onto fresh 1/10 TSA plates and incubated under an N_2_O/N_2_ atmosphere. Single-colony transfer to 1/10 strength TSA plates was repeated two more times before transfer to 160 mL glass serum bottles containing 100 mL of liquid basal salt medium (pH 4.5 and 7) amended with 5 mM fumarate and 416 µmol N_2_O. Following growth, DNA was extracted for PCR amplification using the general bacterial 16S rRNA gene-targeted primer pair 8F (AGAGTTTGATCCTGGCTCAG)−1541R (AAGGAGGTGATCCAGCCGCA) (40) (Integrated DNA Technologies, Inc., [IDT] Coralville, IA, USA), and both strands were subjected to Sanger sequencing. Cell morphology was assessed by light microscopy (1,000-fold magnification, Zeiss Axioscope, Germany). The axenic N_2_O-reducing bacterium was designated strain CFF1 and was routinely grown in 160 mL glass serum bottles containing 100 mL of medium amended with 5 mM fumarate and 4.16 mM (nominal) N_2_O and incubated at 30°C without agitation.

### Construction of a closed circular genome

A metagenomic sequencing library was generated using genomic DNA isolated from a 6th-generation enrichment culture grown at pH 4.5. Sequencing libraries were prepared using the Illumina DNA Prep kit (formerly Nextera DNA Flex Library Prep). Metagenome sequencing was accomplished on the Illumina NovaSeq 6000 platform available at UT’s Genomics Core facility. Metagenomic short reads were processed using the nf-core/mag pipeline (41) (Methods S3) and yielded a MAG with a completeness of 92.2% and less than 5.23% contamination.

To generate a circular genome, PacBio sequencing was performed with DNA extracted from an axenic strain CFF1 culture. The SMRT bell library was sequenced following the PacBio manufacturer’s protocol (Novogene, Durham, NC, USA). PacBio raw reads were corrected using Canu (v2.2) (42), and assembled using Flye v2.9.5 (43). This step generated a single circular contig, which was closed with Circlator v1.5.5 (44). The closed circular contig was corrected with the raw Illumina metagenomic reads generated in 10 rounds/iterations using Polypolish (45). The pairwise average nucleotide identity (ANI) of single-copy genes between the closed genome and fragmented MAG representing strain CFF1 was computed using fastANI (46). ANI analysis demonstrated that the closed circular genome of strain CFF1 matched the fragmented genome (ANI > 99.99%). A circular genome map of strain CFF1 was generated using Proksee (47). Putative horizontally transferred regions were identified using Alien Hunter (48), and insertion sequence elements and integrase genes were detected using mobileOG-db annotations (49). Prophage regions were predicted using PHASTEST (50).

### Reconstruction of NosZ phylogeny

NosZ proteins encoded by *Schinkia* genomes were identified with eggNOG-mapper v2.1.12 (51). Protein sequence-based phylogeny reconstruction of NosZ included 105 clade I, 155 clade II, and 96 clade III NosZ sequences. The NosZ sequences were aligned with MAFFT (52), and the resulting NosZ alignment was trimmed with trimAl v1.4.1 (53). The trimmed alignment was used to create a maximum likelihood protein tree using RaxML-NG v1.2.2 (54). Protein sequence-based tree topology was visualized in iTOL v6 (55).

### Quantitative PCR (qPCR)

A SYBR Green qPCR assay targeting a consensus 16S rRNA gene sequence of strain CFF1 was designed using Geneious Prime version 2024.3.25 (https://www.geneious.com/). The primers (Sch_395F: CACCTTCCTCCGGTTTGTCA; Sch_505R: CAGAGTGACAGGTGGTGCAT) amplified a 200 bp fragment (nucleotide positions 395 to 505). Primer specificity was examined by *in silico* analysis using the Primer-BLAST tool and experimentally confirmed using a linear DNA fragment (IDT) of the consensus 16S rRNA gene of strain CFF1. Enumeration of target 16S rRNA genes was performed as previously described (14) with details provided in the SI (Methods S4). A linear correlation was observed between OD_600 nm_ readings (≤0.15) and qPCR cell enumerations, and OD_600 nm_ measurements were routinely used for rapid estimation of cell numbers.

### Growth yield measurements

Serum bottles (160 mL) with 100 mL of pH 7 medium amended with 5 mM fumarate received 1% (vol/vol) inocula from a fumarate/N_2_O-grown strain CFF1 culture. To determine the growth yields of strain CFF1 with N_2_O as electron acceptor, triplicate cultures received 0, 41.6, 83.2, 208, 416, and 832 µmol of N_2_O. When N_2_O had been completely consumed, OD _600 nm_ readings were recorded before the cells were collected from 1 mL cell suspension samples by centrifugation (13,000 × *g*) for DNA extraction with the DNeasy PowerSoil Kit (Qiagen) and qPCR analysis. For gravimetric growth yield (i.e., cell dry weight) measurements, 90 mL cell suspension samples were collected onto individual polyvinylidene fluoride (PVDF) membrane filters (0.22 µm, 5 cm diameter; Millipore, Billerica, MA, USA) by vacuum filtration (Methods S5). The collected biomass was washed three times with sterile, deionized water to remove residual medium components prior to drying and weighing. Cell dry weight measurements were also performed with O_2_ (91 µmol), NO_3_^−^ (100 µmol), or NO_2_^−^ (100 µmol) provided as electron acceptor.

### Proteomic analysis

Proteomic analysis was performed with strain CFF1 cells grown with N_2_O and fumarate at pH 4.5 and 8.5. Biomass was harvested when at least two-thirds of the initial N_2_O amount had been consumed. Suspension samples (50 mL) were withdrawn, centrifuged, the supernatant was discarded, and the cell pellets were immediately frozen at −80°C. Protein extraction and proteomic analysis followed established procedures (Methods S6). The protein abundance data (i.e., summing of unique peptide abundances) were log_2_-transformed, followed by median centering and LOWESS normalization (56), and then processed in R v4.2.1. Missing values in the raw proteome data were imputed with the “impute.nipal” function implemented in the mixOmics package (57), and a partial least squares discriminant analysis (PLS-DA) model was created. Differential expression analysis, aimed at identifying proteins differentially abundant at pH 4.5 versus 8.5, was performed using the limma package (58).

### Physiological characterization of strain CFF1

Duplicate cultures received 4.16 mM (nominal) N_2_O as electron acceptor and H_2_ (4.16 mM, nominal), formate, acetate, pyruvate, lactate, propionate, succinate, fumarate, butyrate, citrate, or glucose (each at an initial concentration of 5 mM) as potential electron donors. The utilization of alternate electron acceptors, including O_2_ (10 mL air; 0.91 mM O_2_, nominal), NO_3_^−^ (1 mM), NO_2_^−^ (1 mM), and SO_4_^2−^ (1 mM), was tested with 5 mM fumarate as electron donor. The utilization of fumarate as electron acceptor was tested with 5 mM succinate or 5 mM lactate as electron donor. Growth was judged by electron donor and/or electron acceptor consumption and monitoring OD_600 nm_ increases. To examine the denitrification capacity of strain CFF1, triplicate cultures received 2 mM NaNO_3_ or 2 mM NaNO_2_ as electron acceptor. To test the impact of acetylene (99.6%, Airgas, Knoxville, TN, USA) on N_2_O reduction in cultures that received either NO_3_^−^ or N_2_O as the electron acceptor, a volume of 20 mL headspace (33%, vol/vol) was replaced with acetylene. *Stutzerimonas stutzeri* (formerly *Pseudomonas stutzeri*) strain DCP-Ps1, which carries a single clade I *nosZ*, and *Desulfitobacterium dehalogenans* strain DSM9161, which carries a single clade II *nosZ*, cultures served as positive controls for acetylene inhibition. These axenic cultures were grown in pH 7 medium with N_2_O (4.16 mM, nominal) as electron acceptor and 5 mM lactate as electron donor in the presence and absence of 33% (vol/vol) acetylene. No acetylene degradation was observed for any of the axenic cultures under investigation.

To test the response of strain CFF1 to pH, temperature, and salinity, cultures received 5 mM fumarate and 4.16 mM (nominal) N_2_O as substrates. The preparation of media for determining pH, temperature, and salinity ranges is described in the SI (Methods S1). Medium pH ranged from 3.5 to 9.5, and cultures were grown at 30°C. Different temperatures, ranging from 4°C to 50°C, were tested, and cultures were grown in pH 7 medium with 0.1% NaCl. Salinity range was determined by adjusting NaCl concentrations (0.1–3%), and pH 7 cultures were incubated at 30°C. Growth was judged by OD_600 nm_ increases and N_2_O consumption. Cultivation vessels were incubated statically with the stoppers facing up.

### Replication and statistical procedures

Experiments were performed in triplicates, unless stated otherwise. All growth experiments were repeated at least twice in independent experiments. Multivariate analysis was conducted using partial least squares discriminant analysis (PLS-DA) to visualize global differences in protein abundance profiles between experimental conditions. Differential protein abundances between treatments were assessed using the limma package in R (58), which applies linear models with empirical Bayes moderation of variance estimates. Proteins were considered differentially abundant based on an adjusted false discovery rate (FDR)–corrected *P* value < 0.05 and a minimum log_2_ fold-change threshold as indicated in the figure legends. All statistical analyses were performed in R v4.1.2.

### Analytical procedures

N_2_O was analyzed by manually injecting 100 µL headspace samples into an Agilent 3000A Micro-Gas Chromatograph (Palo Alto, CA, USA) equipped with Plot Q and molecular sieve columns coupled to a thermal conductivity detector (59). Headspace N_2_O concentrations were calculated using the ideal gas law. The total volume withdrawn per bottle (9  ×  100  µL) was less than 1.5% of the initial headspace volume (~60  mL); therefore, the associated pressure change was negligible and did not affect the concentration calculations. Aqueous concentrations (µM) of N_2_O were calculated from the headspace partial pressures using the reported Henry’s law constant of 2.4 × 10^−4^ mol (m^3^ Pa)^−1^ (60), according to


(6) $$H^{cp}RT=\frac{C_{a}}{C_{g}}$$

where *H^cp^* is the Henry’s law constant, *R* is the universal gas constant, *T* is the temperature, *C_g_* is the headspace gas phase concentration, and *C_a_* is the liquid phase (dissolved) molar concentration. Five-point standard curves for N_2_O spanned a concentration range of 8,333 to 133,333 ppmv. Fumarate, acetate, formate, pyruvate, citrate, and succinate were measured with an Agilent 1200 Series high-performance liquid chromatography (HPLC) system (Palo Alto, CA, USA) as previously described (59). Optical density (OD) was measured with an automated plate reader at 600 nm (Synergy 2, BioTek, Winooski, VT, USA).

## RESULTS

### Enrichment and isolation of a novel N_2_O-reducing bacterium

Anoxic microcosms derived from CFF soil reduced repeated feedings of N_2_O (~1.2 mmol total) to N_2_ at pH 4.5 with fumarate provided as electron donor over a 2-week incubation period. N_2_O reduction activity was maintained in transfers to fresh medium with the medium pH alternating from pH 4.5 to pH 7 to pH 4.5 between six consecutive transfers (Fig. 1A). This pH-alternating enrichment strategy resulted in a mixed culture with robust N_2_O reduction activity at pH 4.5 and at pH 7 (Fig. 1B and C). 16S rRNA gene amplicon sequencing of DNA isolated from the enrichment culture grown with fumarate and N_2_O at pH 4.5 and at pH 7 demonstrated that a 426 bp long *Bacillus*-related amplicon sequence variant (ASV) accounted for over 60% of total 16S rRNA gene ASVs under both pH conditions (Fig. 1D and E). Plating of diluted culture suspension samples on 1/10- and full-strength TSA plates followed by a 48-hour incubation inside a gas-tight jar under an N_2_:N_2_O (9:1, vol:vol) atmosphere or an N_2_ atmosphere without N_2_O yielded uniform, round, opaque colonies with undulate margins ranging from 0.2 to 0.3 mm in diameter. Streak plating yielded uniform colonies, and incubation under an N_2_:N_2_O atmosphere was repeated twice before four individual colonies were transferred to separate vessels containing anoxic, defined basal salt liquid medium (pH 7) amended with fumarate and N_2_O. Growth and N_2_O consumption occurred in all inoculated vessels, and transfer cultures grew with N_2_O as electron acceptor in pH 4.5 and in pH 7 medium over a 5-day incubation period. Sanger sequencing determined nearly complete (1,444–1,463 bp) 16S rRNA gene sequences with > 99.7% identity, suggesting that the colonies represent the same bacterial population. The complete genome of the isolate (see below) encodes 15 16S rRNA gene variants of >99.5% sequence identity, which showed ≥99.1% identity to the Sanger sequencing-derived 16S rRNA gene sequences, corroborating culture purity (Fig. S1). Phase contrast light microscopy revealed motile, rod-shaped cells of uniform morphology, approximately 5 µm long and 0.2 µm wide (Fig. 1A). Growth also occurred under oxic conditions with identical colony shape and cell morphology observed on agar plates and in liquid medium, respectively. The results from cultivation, visual observation, and sequence analyses all support culture purity, and the isolate was designated strain CFF1.

### N_2_O reduction by strain CFF1

**Fig 2:**
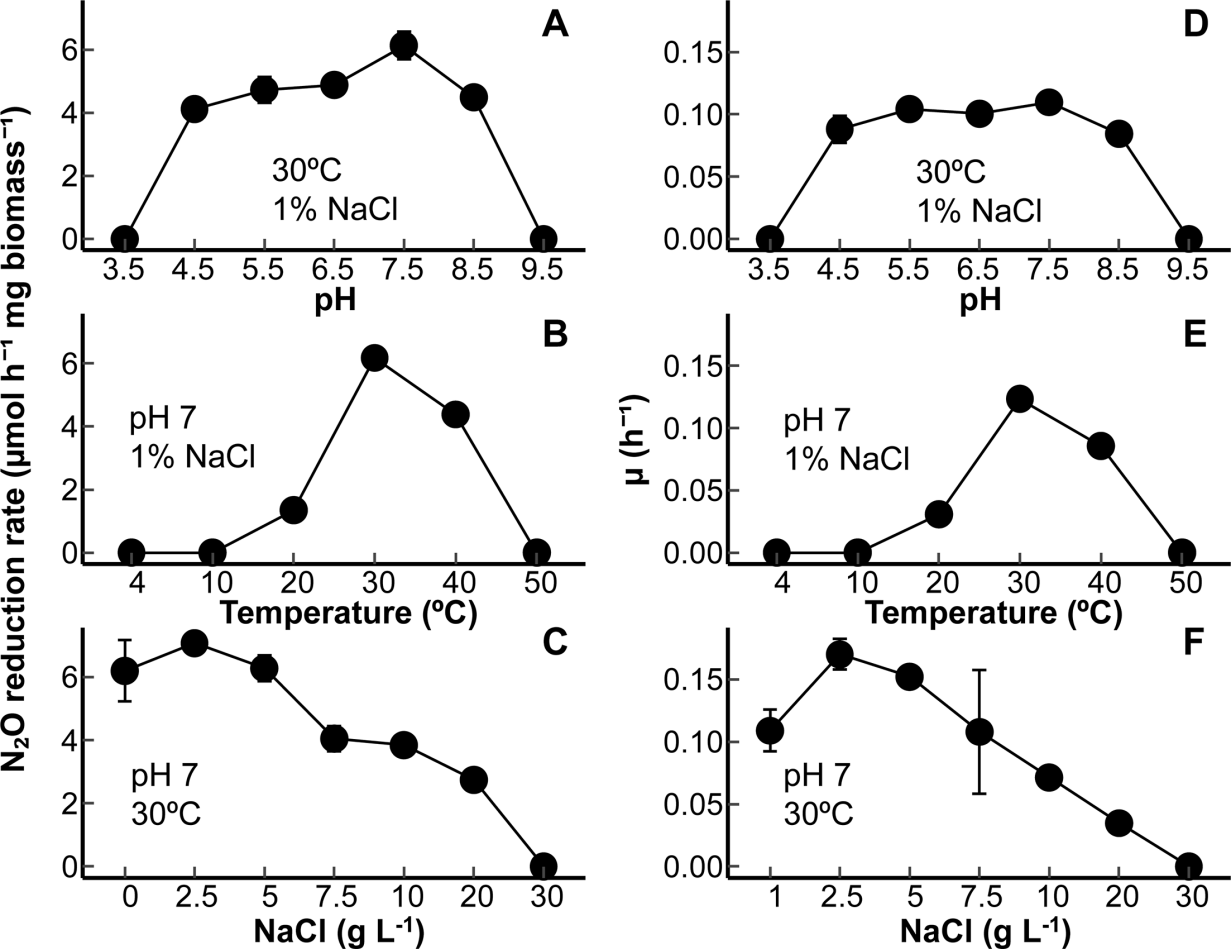
Effects of pH, temperature, and salinity on N_2_O reduction and growth of strain CFF1. Panels **A**, **B**, and **C **on the left show effects of pH, temperature, and salinity, respectively, on N_2_O reduction rates, and panels **D**, **E**, and **F** depict the effects on growth rates of strain CFF1. No N_2_O reduction activity was observed over a 2-month incubation period at pH 3.5 and 9.5, at 4°C, 10°C, and 50°C, and at salinities exceeding 3% (wt/vol) of NaCl. The specific growth rates (μ) were determined by regression of ln(OD _600 nm_) against time during the period of exponential growth. Exponential growth phases were identified by monitoring optical density (OD_600 nm_) at regular intervals and selecting the time window in which OD _600 nm_ values increased linearly on a logarithmic scale. Unless indicated otherwise, the culture vessels were incubated statically at 30°C and pH 7 with 0.1% (wt/vol) NaCl in the standard growth medium. The data represent averages of triplicate cultures, and the error bars depict the standard deviations. Where error bars are not visible, they are smaller than the symbols.

In laboratory cultivation experiments, strain CFF1 reduced N_2_O over a broad pH range spanning pH 4.5 to 8.5 (Fig. 2), with no N_2_O consumption or growth observed at pH 3.5 and pH 9.5. Cell densities during N_2_O-dependent growth remained below an OD_600 nm_ value of 0.2, with no visible cell aggregation observed by light microscopy. Under the growth conditions tested, the medium pH changes during the cultivation periods were small (<0.5 pH units). pH impacted the onset of growth and N_2_O consumption, with apparent lag phases of ~12 h observed at pH 4.5, 5.5, and 8.5, and shorter lag phases of ~6 h at pH 6.5 and 7.5. The highest N_2_O reduction rates of 6.13 ± 0.30 µmol N_2_O h^−1^ mg biomass^−1^ (dry weight) were observed at pH 7.5, with 33 ± 5 and 27 ± 2% lower activity measured at pH 4.5 and 8.5, respectively (Fig. 2A). Similar growth rates (µ) of 0.10 ± 0.01 h^−1^ were determined between pH 5.5 and 7.5, with slightly lower µ values observed at pH 4.5 and 8.5 (Fig. 2D). The highest N_2_O reduction rates and the highest growth rates were observed at 30°C (Fig. 2B and E). Growth and N_2_O reduction occurred at 20°C and 40°C, but growth or N_2_O reduction was not apparent at 4°C, 10°C, or 50°C over a 2-month incubation period. Salinity impacted growth and N_2_O reduction, and the highest rates were measured with 2.5 g NaCl L^−1^ added to the basal salt medium, with rate reductions of 13% and 61% observed with 1 and 20 g L^−1^ NaCl, respectively (Fig. 2C and F). Neither growth nor N_2_O reduction occurred with 30 g L^−1^ NaCl over a 2-month incubation period.

### Taxonomic classification of the *Bacillus*-related bacterium

The complete 4,236,937 bp genome (comprising both the origin and the terminus of replication) harbors 16 5S, 15 16S, and 15 23S rRNA genes (Table S1). 16S rRNA gene sequence analysis affiliated strain CFF with the genus *Schinkia* (formerly *Bacillus*) (61). Genome-based taxonomic placement corroborated this assignment, and strain CFF1 was most closely related to *Schinkia oryziterrae* strain ZYK and *Calidifontibacillus erzurumensis* strain P2 (Fig. S2). Based on pairwise genomic average nucleotide identity (ANI) and digital DNA-DNA hybridization (dDDH) analysis, strain CFF1 displays 78.3–80.0% ANI and 14.4–20.7% dDDH, respectively, to related *Schinkia* species, including *Calidifontibacillus erzurumensis*. These similarity indices are well below the species delineation thresholds of 95% ANI and 70% dDDH (62, 63), indicating that strain CFF1 represents a novel *Schinkia* species. Members of *Schinkia* genus can be distinguished from other *Bacillaceae* species by 13 conserved signature indels (CSIs) (61). The strain CFF1 genome contains 12 of the 13 CSIs shared by *Schinkia* spp., and a gene encoding the ammonia-forming cytochrome *c* nitrite reductase subunit c552 is absent on strain CFF1 genome (Table S2).

### Physiological features of *Schinkia* sp. strain CFF1

Strain CFF1 grew in a defined, completely synthetic basal salt medium by coupling N_2_O reduction with the oxidation of fumarate, succinate, lactate, pyruvate, or propionate (Table S3). No growth was observed with glucose, citrate, butyrate, acetate, formate, or hydrogen (H_2_) in the presence or absence of N_2_O. Aerobic growth with glucose, citrate, acetate, formate, butyrate, or hydrogen did not occur (data not shown).

**Fig 3:**
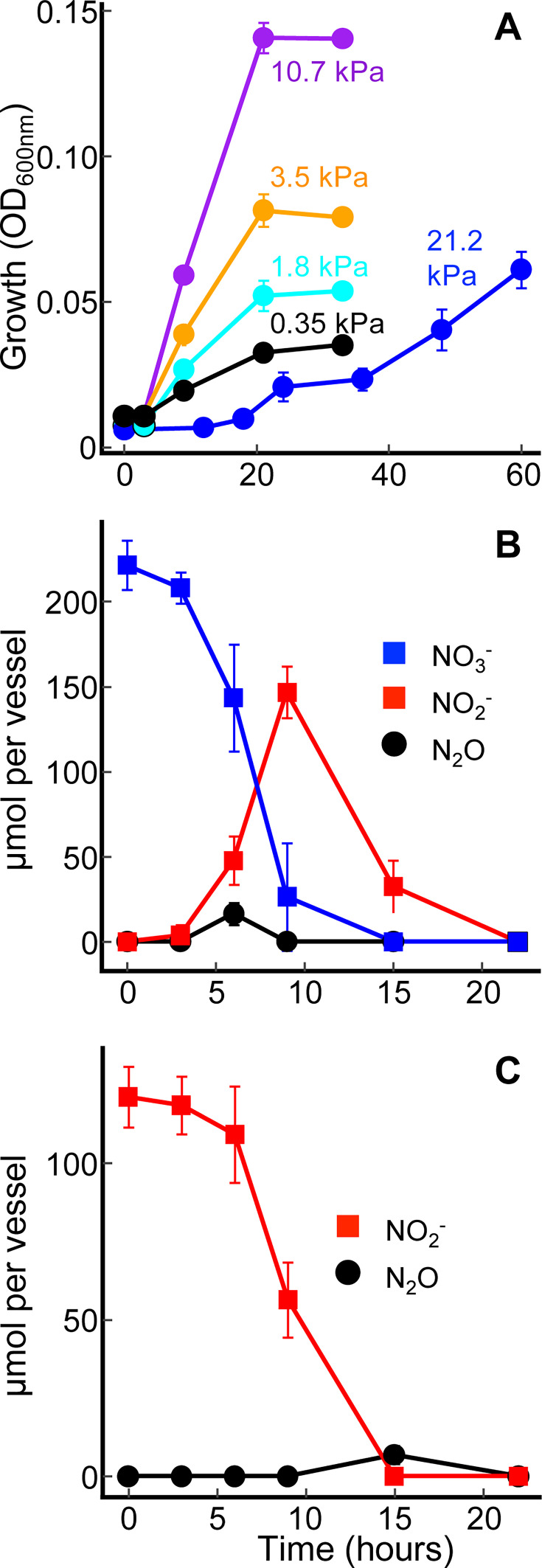
Growth of *Schinkia acidoalkalinis* strain CFF1 with O_2_, NO_3_^−^, or NO_2_^−^ as an electron acceptor. Panel **A** shows growth of strain CFF1 at initial O_2_ partial pressures ranging from 0.35 to 21.2 kPa (i.e., atmospheric O_2_). Panel **B** depicts consumption of NO_3_^−^ with intermittent formation of NO_2_^−^ and N_2_O. Panel **C **shows the consumption of NO_2_^−^ provided as an electron acceptor. The data represent averages of triplicate cultures, and the standard deviations depict performance variations. Error bars are not shown when smaller than the symbols.

With fumarate as electron donor under ambient air (21.2 kPa O_2_), growth commenced following an ~18-hour lag time. Substantially shorter lag periods were observed in replicate cultures that experienced lower O_2_ partial pressures of 0.35–10.7 kPa, suggesting that strain CFF1 prefers microoxic conditions (Fig. 3A). In addition to O_2_ and N_2_O, strain CFF1 utilizes nitrate (NO_3_^−^) as an electron acceptor and performs complete denitrification (NO_3_^−^→ N_2_), with transient formation of nitrite (NO_2_^−^) and N_2_O (Fig. 3B). NO_2_^−^ (1 mM) also supports growth as an electron acceptor (Fig. 3C). Sulfate was not utilized as an electron acceptor with fumarate or succinate as electron donors (data not shown). No growth, fumarate consumption, and succinate formation were observed in cultures that received fumarate as an electron acceptor with propionate or lactate provided as the electron donor, indicating strain CFF1 does not respire fumarate. Electron donor consumption, including fumarate and succinate, strictly depended on a growth-supporting electron acceptor, such as N_2_O. Cultures that received N_2_O as an electron acceptor consumed fumarate or succinate in stoichiometries consistent with complete oxidation to CO_2_ (Table 2; Fig. S3).

**TABLE 2:** Stoichiometry of fumarate and succinate oxidation coupled to N_2_O consumption and calculation of electron recovery in axenic strain CFF1 cultures

Electron donor	Electron donor consumed (μmol vessel^−1^)	Electrons released (μmol vessel^−1^)^*a*^	N_2_O consumed (μmol vessel^−1^)	Electrons consumed (μmol vessel^−1^)^*b*^	Electron balance (%)
Fumarate	89.3 ± 0.71	1,071.6 ± 8.52	400 ± 9.91	800 ± 19.8	75 ± 0.02
Succinate	67.0 ± 1.25	938 ± 17.5	404 ± 6.91	808 ± 13.8	86 ± 0.01

^*a*^ Assuming complete oxidation to CO_2_ as evidenced by the lack of organic acid (e.g., acetate) formation and the calculated demand of reducing equivalents for N_2_O reduction (N_2_O + 2[H] → N_2_ + H_2_O). Electron donors were added in at least 2-fold excess.

^*b*^ Electron consumption was calculated based on the theoretical electron demand according to N_2_O + 2H^+^ + 2e^-^→ Ν_2_ + H_2_O.

Based on the observed stoichiometries, strain CFF1 couples the oxidation of fumarate or succinate with N_2_O reduction according to:


$$C_{4}H_{4}O_{4}\;+6N_{2}O\to 4\;CO_{2}+\;6\;N_{2}\;+2\;H_{2}O$$

$$C_{4}H_{6}O_{4}\;+\;7\;N_{2}O\to 4\;CO_{2}\;+7\;N_{2}\;+3\;H_{2}O$$

The addition of acetylene to the cultivation vessel headspace (33%, vol/vol), a known inhibitor of N_2_O reductase (NosZ) activity, did not inhibit N_2_O reduction by strain CFF1 during denitrification with 1 mM NO_3_^−^. Similarly, acetylene did not prevent N_2_O reduction and growth of strain CFF1 with N_2_O as an electron acceptor (Fig. S4). In control experiments, *Stutzerimonas stutzeri* and *Desulfitobacterium dehalogenans* respired N_2_O with lactate as an electron donor, but the addition of acetylene abolished N_2_O reduction, highlighting the distinct response of strain CFF1 to acetylene.

Growth yields, expressed as mg biomass (dry weight) produced per mmol of electron acceptor consumed, were determined for different respiratory conditions, with fumarate provided as an electron donor. The highest growth yield was measured with NO_3_^−^ as an electron acceptor, with 49.5 ± 4.4 mg biomass produced per mmol NO_3_^−^ consumed. Growth yield reductions of 42%, 79%, and 27% were observed with NO_2_^−^, O_2_, or N_2_O as electron acceptors (Table 3). The highest growth rate of 0.16 ± 0.3 h^−1^ was measured with O_2_ as an electron acceptor in pH 7 medium at 30°C, with 18%, 44%, and 44% lower growth rates observed with N_2_O, NO_3_^−^, or NO_2_^−^ as electron acceptors, respectively.

**TABLE 3:** Growth kinetic parameters of strain CFF1 determined in defined basal salt medium (pH 7) with 5 mM fumarate as an electron donor and O_2_, NO_3_^−^, NO_2_^−^, or N_2_O as the sole electron acceptor at 30°C^*a*^

Electron acceptor (EA)^*b*^	Reduction half reaction	Growth yield (mg dry weight)		µ (h^−1^)^*c*^
		mmol of EA reduced^−1^	mmol of e^−^ transferred to EA^−1*d*^	
O_2_	O_2_ + 4H^+^ + 4e^−^→ 2H_2_O	36.0 ± 1.81^*e*^	8.99 ± 0.45	0.16 ± 0.3
NO_3_^–^	2NO_3_^−^ + 12H^+^ + 10e^−^→ N_2_ + 6H_2_O	49.5 ± 4.40^*f*^	9.90 ± 0.88	0.09 ± 0.02
NO_2_^–^	2NO_2_^−^ + 8H^+^ + 6e^−^→ N_2_ + 4H_2_O	28.3 ± 1.01^*g*^	9.42 ± 0.34	0.09 ± 0.02
N_2_O	N_2_O + 2H^+^ + 2e^−^→ N_2_ + H_2_O	10.2 ± 2.38	5.08 ± 1.19	0.13 ± 0.01

^*a*^ The data represent averages of triplicate cultures, and the standard deviations depict variations between replicates.

^*b*^ Total amounts of 93 µmol O_2_, 110 µmol NO_3_^−^, 110 µmol NO_2_^−^ , or 405 µmol N_2_O were completely reduced. Electrons transferred to each electron acceptor were calculated based on the reduction half reactions.

^*c*^ Maximum observed growth rate based on OD_600 nm_ measurements.

^*d*^ Calculations of growth yield (mg dry weight) per mmol of electrons (e^−^) consumed in electron acceptor (EA) reduction are based on the observation that the electron donor fumarate was oxidized to CO_2_ (Table 2).

^*e*^ *P *value of 0.0002 compared to N_2_O (two-sided Welch’s *t*-test).

^*f*^ *P *value of 0.0005 compared to N_2_O (two-sided Welch’s *t*-test).

^*g*^ *P *value of 0.0011 compared to N_2_O (two-sided Welch’s *t*-test).

### Comparative genomic analyses

**Fig 4:**
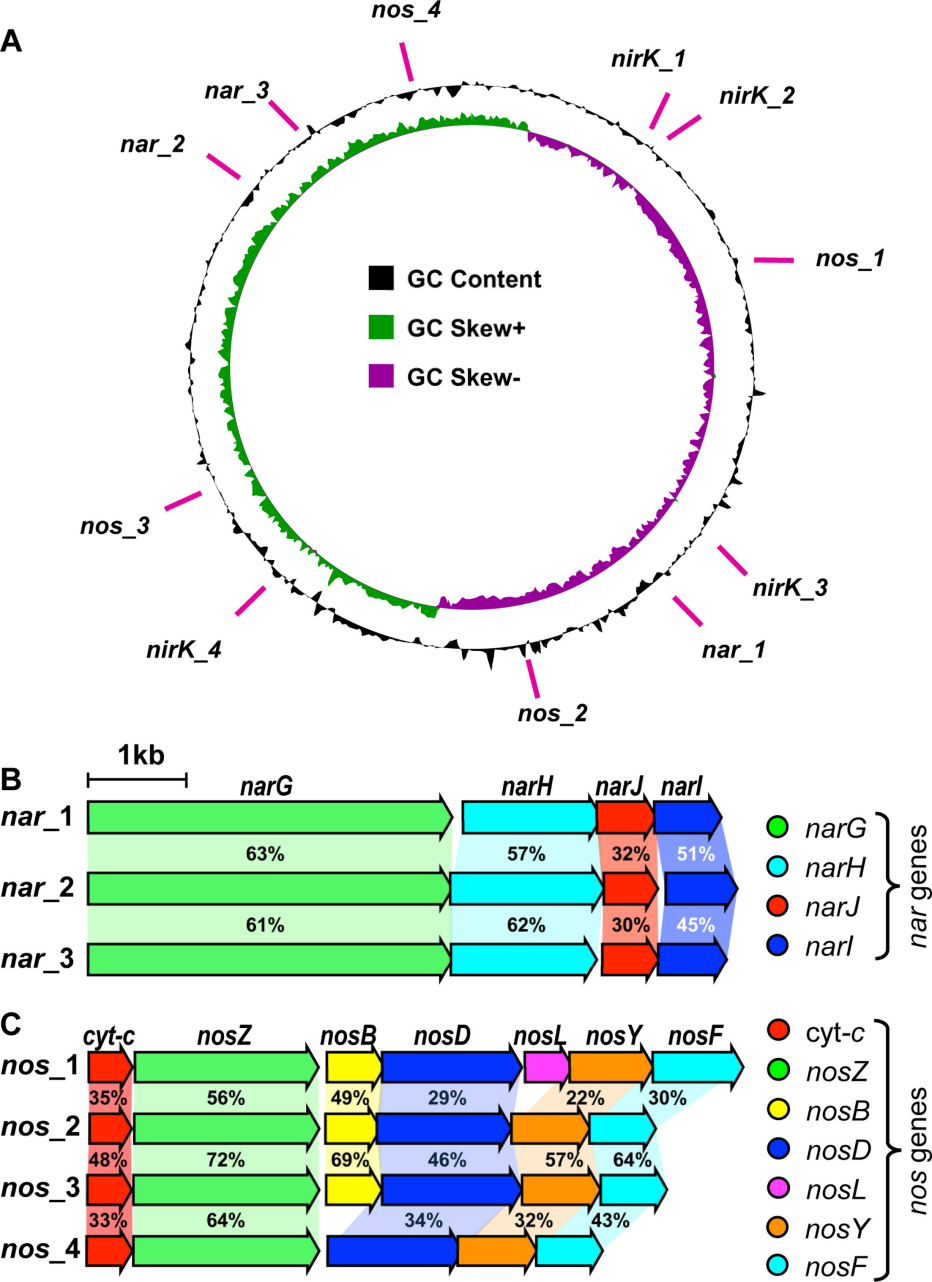
The *Schinkia acidoalkalinis* strain CFF1 genome and gene clusters encoding NO_3_^−^ and N_2_O reductases. Panel **A **shows a circular map of the strain CFF1 genome, with the inner circle indicating the GC skew and the outer circle showing the GC content. Panel **B **depicts the gene arrangements in the three *nar* gene clusters encoding NO_3_^−^ reductases. The arrows indicate *narG* (green), *narH* (cyan), *narJ* (red), and *narI* (blue) genes. Panel **C **shows the gene arrangements in the four *nos* gene clusters encoding clade II N_2_O reductases. The scale bar serves as an estimator of gene length. The shaded areas with percentage values indicate AAI between homologous protein sequences.

To assess denitrification potential in bacteria closely related to strain CFF1 (Table S1), a comparative genomic analysis was conducted. *Schinkia* sp. strain CFF1 harbors three *narG* genes encoding NO_3_^−^ reductases and four clade II *nosZ* genes (Fig. 4A). Three *nar* gene clusters (i.e., *nar*_1–3), each comprising *narGHJI* characteristic of Nar-type NO_3_^−^ reductases, were identified (Fig. 4B). The three *nar* gene clusters share the same gene order (synteny) but are divergent, with average amino acid identity (AAI) values ranging from 30 to 63% between individual *nar* genes (i.e., *narGHJI*). The *Schinkia* sp. strain CFF1 genome also harbors four *nirK* genes encoding NO_2_^−^ reductases with AAI ranging from 72 to 95%. Four non-identical NosZ-encoding *nos* gene clusters, referred to as *nos*_1–4, are present on the strain CFF1 genome (Fig. 4C). Only *nos*_1 comprises all of the known *nos* accessory genes (i.e., *nosBDLYF*) characterized for their roles in NosZ maturation and function (64), while three of the *nos* gene clusters lack *nosL*. Cluster *nos*_4 lacks *nosB*, a characteristic gene of clade II *nos* clusters that encodes a transmembrane protein (12). The highest AAI of 72% was observed for NosZ, while the NosY protein has the lowest AAI (22%). Interestingly, NosZ_1 lacks recognizable Sec and Tat signal peptides, while NosZ_2–4 harbor Sec signal peptides at the N-terminus characteristic for clade II.

Comparative genomic analysis revealed substantial variation in denitrification gene repertoire across the 26 *Schinkia* and the single *Calidifontibacillus* genomes (Table S1). All *S. azotoformans* genomes encode three *nar* (except the strain LMG 9581 genome with two *nar* genes), 3–4 *nos* gene clusters, and a single *nirK* gene (Table S1). The *S. oryziterrae* genome contains one *nar* and two *nos* gene clusters, but does not possess *nirK* or *nirS* (Fig. S5), suggesting that this bacterium cannot denitrify. The presence of multiple, non-identical *nos* gene clusters is a distinguishing feature of *Schinkia*, and the available genomes representing other genera in the *Bacilliaceae* family harbor no more than one *nos* gene cluster. Notably, *nosZ* genes from the same *Schinkia* genome showed greater sequence divergence from each other than *nosZ* genes from other *Schinkia* species (Fig. S6). The *nos* gene clusters did not overlap with regions predicted as horizontally transferred, and insertion sequences and integrase or prophage-related genes were not detected adjacent to *nos* genes. Taken together, these observations suggest that allelic variation or horizontal transfer of *nosZ* genes occurred but likely preceded speciation.

Characterized *S. azotoformans* strains (*n* = 3) have been reported to utilize fumarate as respiratory electron acceptor in medium supplemented with 4 g L^−1^ yeast extract (65), while strain CFF1 uses fumarate exclusively as an electron donor in defined basal salt medium. Consistently, *frdAB* genes encoding respiratory fumarate reductase were not found on the strain CFF1 genome, and *S. azotoformans* and *S. oryziterrae* also lack *frdAB* genes. *Schinkia* species have been reported to be spore formers (65, 66), and the genome of strain CFF1 harbors at least 38 sporulation genes. The genomes of spore-forming *S. azotoformans* and *S. oryziterrae* encode 36–43 genes associated with sporulation.

Preferential growth conditions (e.g., temperature, pH, and salinity) of *Schinkia* strains were predicted using genome-based computational models (67). The analysis of 26 *Schinkia* genomes predicted similar optima for temperature (41.0 ± 0.82°C), pH (7.64 ± 0.11), and salinity (2.00 ± 0.32%). Notably, the predicted minimum pH of 5.53 for growth of strain CFF1 was lower than the pH values predicted for other *Schinkia* species (typically ≥ 6.0) and aligned with its acid-tolerant phenotype. The model-predicted pH range for growth of strain CFF1 was 5.53–9.25 (Fig. S7A), about 1 pH unit higher than the experimentally observed range of 4.5–8.5 (Fig. 4A). The model-predicted minimum salinity requirement was 1.98% (Fig. S7B), but growth was observed in the standard basal salt medium containing 0.1% NaCl. The model-predicted temperature range for growth was 19.7°C to 48.4°C (Fig. S7C), and the observed temperature growth range of characterized *Schinkia* spp., including strain CFF1, is 20°C to 40°C.

Collectively, the physiological and genomic features indicate that the strain CFF1 represents a novel species of the genus *Schinkia*. Based on the unique ability for N_2_O reduction at acidic and alkaline pH, we propose the name *Schinkia acidoalkalinis* strain CFF1 (a.ci.do.al.ka.li’nis. L. pref. acido-, pertaining to acid; L. adj. alkali, pertaining to alkaline; N.L. masc./fem. n. acidoalkalinis, an organism capable of N_2_O reduction in both acidic and alkaline environments). The strain designation CFF1 refers to Caney Fork Farms, a farm practicing regenerative agriculture in Carthage, TN, USA, from where the soil for establishing microcosms was collected.

### Proteome response of *Schinkia acidoalkalinis* strain CFF1 to external pH

**Fig 5:**
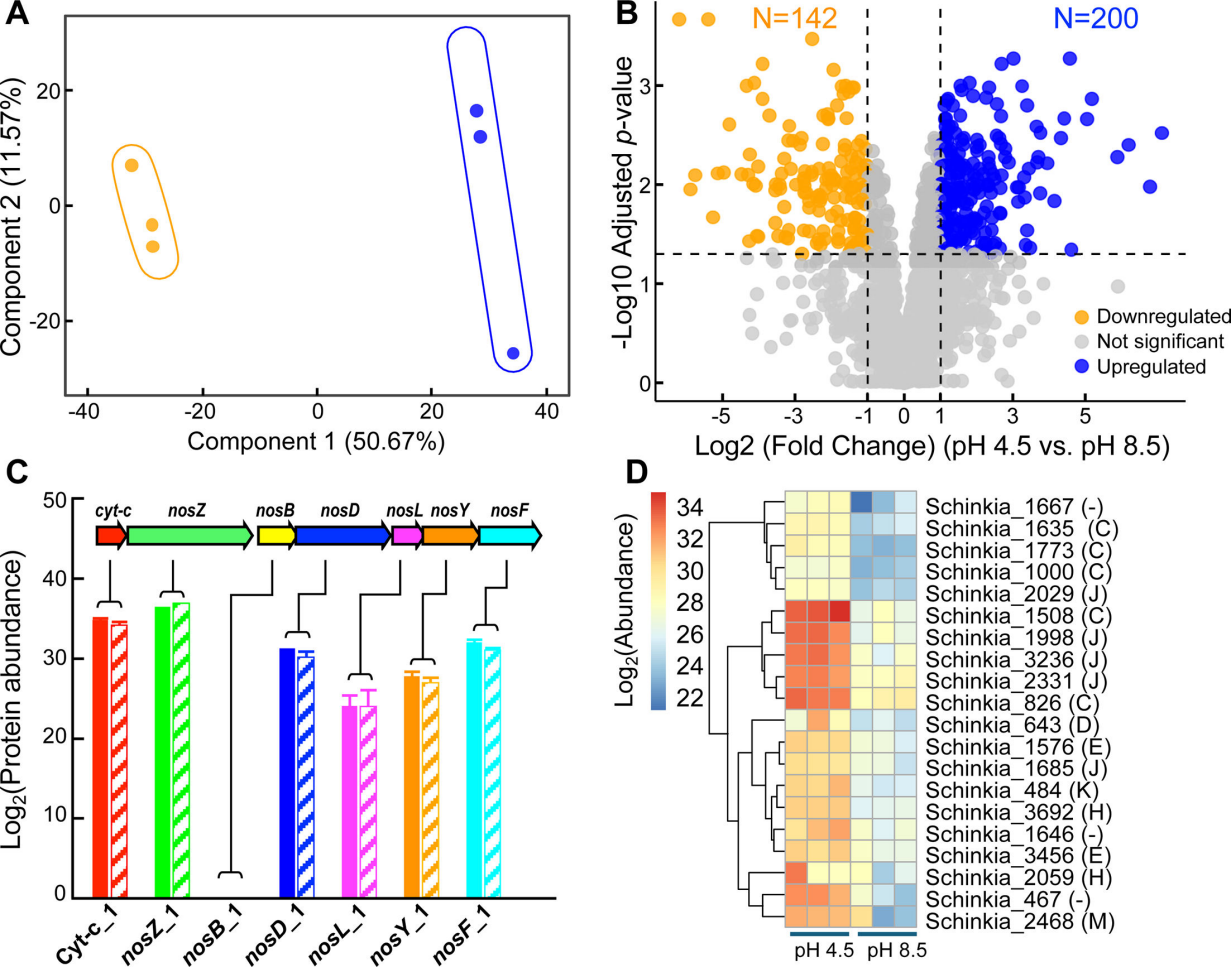
Proteome response of *Schinkia acidoalkalinis* strain CFF1 to external (i.e., medium) pH. Panel **A **shows a PLS-DA plot of proteomes of strain CFF1 cells grown at pH 4.5 versus 8.5. The PLS-DA indicates distinct clustering (95% confidence) of proteomes based on medium pH. Each datum point represents a proteome generated from an independent culture replicate. Ellipses represent the 95% confidence regions around the group centroids in the PLS-DA score plot. Panel **B** depicts a volcano plot showing the pairwise comparison of proteins (datum points in different colors) in strain CFF1 cultures grown at pH 4.5 (orange circles) versus pH 8.5 (blue circles). The abundance fold change was determined based on detected protein abundances under the respective pH growth conditions. Criteria for calling protein abundance significantly up- or downregulated included log_2_(fold change) >1 and log_2_(fold change) < −1, respectively, and adjusted *P* values of < 0.05. Proteins not meeting these criteria were considered to not undergo significant abundance changes in pH 4.5 versus 8.5 growth conditions. The numbers in blue and orange font indicate the number of upregulated proteins at pH 4.5 and 8.5, respectively. Panel **C** depicts a bar plot showing log_2_-transformed abundance of Nos_1 proteins in strain CFF1 cultures grown at pH 4.5 (solid bars) and 8.5 (striped bars). The heatmap (**D**) shows the abundance of the top 20 differentially abundant proteins in cells grown at pH 4.5 versus pH 8.5. The letters in parentheses refer to the top-level categories in the Cluster of Orthologous Groups (COGs) database: (C) energy production and conversion; (E) amino acid transport and metabolism; (J) translation, ribosomal structure, and biogenesis; (D) cell cycle control, cell division, chromosome partitioning; (H) coenzyme transport and metabolism; (K) transcription; (M) cell wall/membrane/envelope biogenesis; and (−) proteins not assigned to any top-level categories in the COG database. The results represent data from triplicate cultures.

Proteomic analyses were conducted to investigate the effect of pH on NosZ synthesis. Comparative proteomic analyses revealed distinct abundance profiles in strain CFF1 cells grown at pH 4.5 versus pH 8.5. A total of 2,156 proteins were identified across all samples (Table S4), with 59 and 37 proteins exclusively detected in cells collected from pH 4.5 versus pH 8.5 grown cultures, respectively (Table S5). PLS-DA showed distinct clustering (95% confidence) of proteomes based on the medium pH (Fig. 5A). Protein-level differential abundance analysis with limma (58) revealed that 200 and 142 individual proteins were more abundant (fold change > 2, Wald test with Benjamini-Hochberg adjusted *P* < 0.05) in pH 4.5 versus pH 8.5 samples, respectively (Fig. 5B). In strain CFF1, two (i.e., NosZ_1 and NosZ_3) of the four NosZ proteins were detected at similar abundance levels when grown in pH 4.5 and in pH 8.5 medium (Fig. 5C). The auxiliary proteins NosDLYF and a *c*-type cytochrome juxtaposed to NosZ_1 were all detected, but no peptides of the transmembrane protein NosB were observed. The auxiliary proteins associated with NosZ_3 were detected in one of the three biological replicates for each pH group. Peptides representing NosZ_2 or NosZ_4, or any of the associated auxiliary proteins, were not detected under either pH growth condition. The abundance of proteins involved in energy production and conversion (COG category C) and in translation, ribosomal structure, and biogenesis (category J) had higher detected abundance, at least 8-fold in strain CFF1 cultures grown at pH 4.5 versus pH 8.5 (Fig. 5D). These findings highlight strain CFF1’s adaptive changes in response to medium pH; however, the measurements suggest that pH does not alter NosZ expression.

### Metabolic growth model with N_2_O as electron acceptor

**Fig 6:**
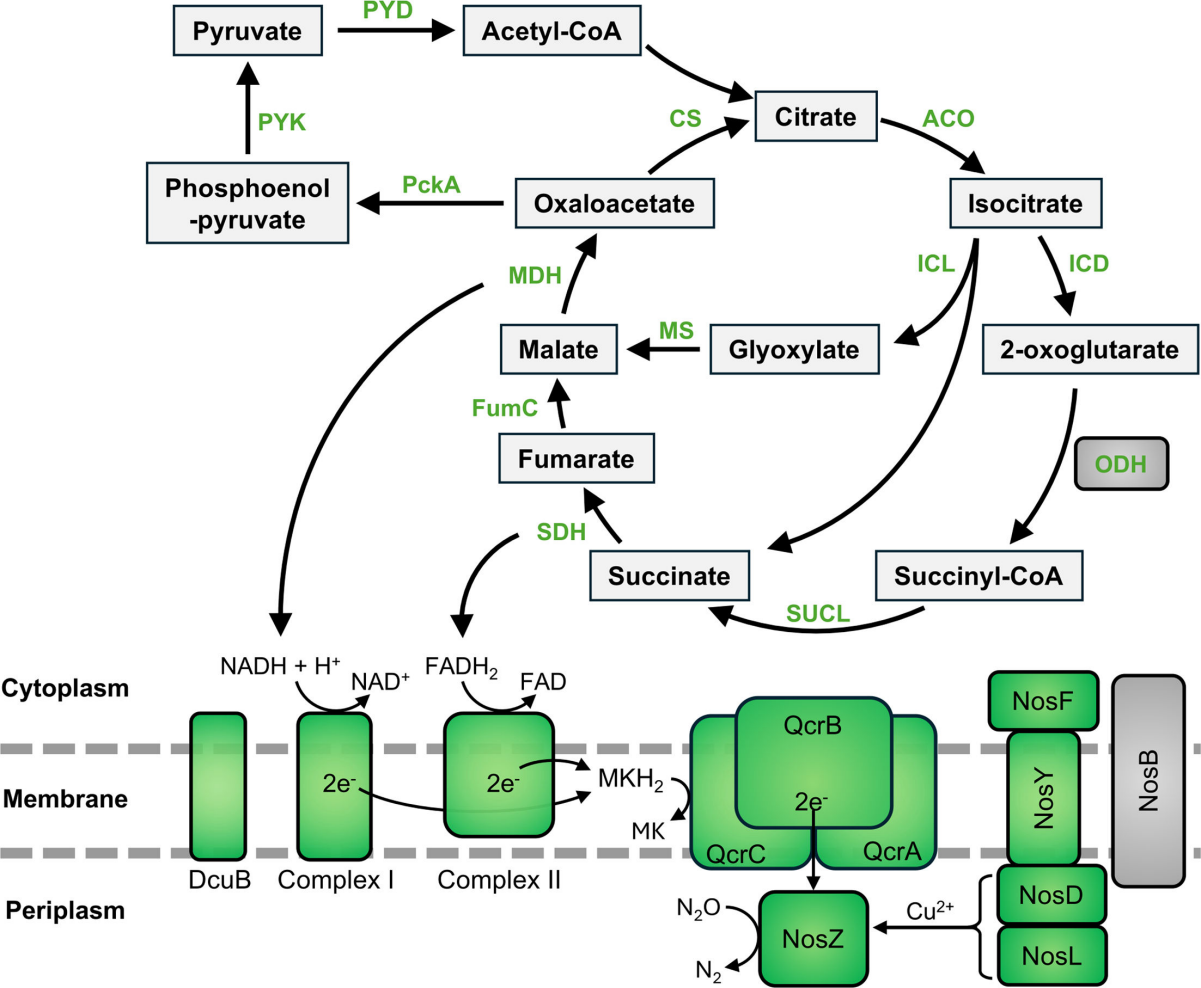
Gene content- and protein abundance-based metabolic model coupling fumarate oxidation with N_2_O reduction observed in *Schinkia acidoalkalinis* strain CFF1. DcuB, anaerobic C4-dicarboxylate (e.g., fumarate, succinate) transporter. FumC, fumarate hydratase; MDH, malate dehydrogenase; CS, citrate synthase; ACI, aconitate hydratase; ICD, isocitrate dehydrogenase; ODH, 2-oxoglutarate; SCS, succinyl-CoA ligase; SDH, succinate dehydrogenase; ICL, isocitrate lyase; MDH, malate dehydrogenase; PEPCK, phosphoenolpyruvate carboxykinase; PK, pyruvate kinase; PD, pyruvate dehydrogenase; QcrABC form a cytochrome bc1 complex; NosZ, N_2_O reductase; NosBDLYF, auxiliary proteins essential for NosZ maturation and function. Proteins in green are detected in biomass grown with fumarate and N_2_O. Proteins in gray are encoded on the strain CFF1 genome but not detected during growth with N_2_O. Electron flow is based on prior findings (69).

Integrated genome and proteome analyses generated a predictive metabolic model that captures pathways active in strain CFF1 during growth with the fumarate/N_2_O redox couple. All essential genes involved in the conversion of pyruvate to acetyl-CoA and in the TCA cycle were present on the strain CFF1 genome, and the respective proteins were detected under acidic and alkaline growth conditions (Fig. 6). A notable exception was the 2-oxoglutarate dehydrogenase complex, ODH, which is encoded on the strain CFF genome, but OdhA and OdhB proteins were not detected at either pH growth condition. Instead, isocitrate lyase (AceA), which catalyzes the conversion of 2-oxoglutarate to succinate and glyoxylate (68), was detected in the strain CFF1 proteomes. This observation suggests that strain CFF1 bypasses the conversion of isocitrate to 2-oxoglutarate and subsequently to succinate, presumably operating a glyoxylate shunt. Also detected were the three subunits QcrA, QcrB, and QcrC of a cytochrome bc1 complex, an essential component of the electron transport chain in N_2_O respiration (69).

## DISCUSSION

### *Schinkia acidoalkalinis* strain CFF1 is an N_2_O-respiring pH generalist

The interaction between pH and N_2_O-respiring microorganisms plays a crucial role for N_2_O emissions from soil ecosystems. High N_2_O fluxes and emission factors (i.e., the proportion of N fertilizer lost in the form of N_2_O) have been reported for acidic soils (i.e., pH < 6) (70), presumably due to impaired activity of neutrophilic N_2_O reducers (16–18). This paradigm has been revised, and recent findings describe acidophilic bacteria that respire N_2_O under acidic pH conditions (i.e., pH 2–6) (27, 28, 35). However, the acidophilic bacteria described to date lose N_2_O-reducing activity above pH 6.5 (27, 35). These findings suggest niche specialization with distinct bacterial taxa performing N_2_O reduction in acidic versus alkaline pH environments.

We applied a dual pH enrichment strategy with the goal of selecting for microorganisms capable of N_2_O reduction over a broader pH range. This enrichment strategy was recently introduced to obtain N_2_O-reducing bacterial isolates with generalist lifestyles in terms of growth in digestate and soil (34). In our study, enrichment through sequential transfers to medium with alternating pH (i.e., pH 4.5 → pH 7 → pH 4.5) yielded an enrichment culture that reduced N_2_O at similar rates at pH 4.5 and 8.5. Ultimately, a novel facultative denitrifying bacterium, *Schinkia acidoalkalinis* strain CFF1, was isolated that sustains growth via N_2_O respiration between pH 4.5 and 8.5, indicating that N_2_O-reducing pH generalists exist in agricultural soils. The pH-alternating sequential transfer procedure selected for a soil bacterium capable of N_2_O-reduction over a broad pH range, highlighting the utility of the dual enrichment strategy.

### A novel denitrifying *Schinkia* species

Members of the genus *Schinkia* (formerly *Bacillus*) are strict aerobes or facultatively anaerobes (61, 65, 66). Strain CFF1 grows with O_2_, NO_3_^−^, NO_2_^−^, or N_2_O as the sole electron acceptor and is classified as a facultative denitrifying bacterium. All characterized *Schinkia* spp. perform complete denitrification at circumneutral pH (65, 66), with no growth observed below pH 6, consistent with genome-based predictions, suggesting a lower pH threshold of ~6.0. Strain CFF1 does not follow this pattern and represents the first low pH-tolerant *Schinkia* isolate. While the GenomeSPOT analysis (67) of the strain CFF1 genome predicted a lower pH tolerance limit of 5.6, experimental efforts showed that strain CFF1 grows via N_2_O respiration at pH 4.5. The genome-based prediction suggests that strain CFF1 only grows with NaCl concentrations of 20 to 65 g L^−1^, while the experimental work demonstrated growth between 1 and 20 g L^−1^ NaCl. These discrepancies between computational prediction and actual measurements highlight the limitations of genome-based ecological inference models and the importance of experimental validation for determining microbial responses to environmental factors.

Characterized *Schinkia azotoformans* (formerly *Bacillus azotoformans*) strains were reported to utilize fumarate as an electron acceptor with 4 g L^−1^ yeast extract as an electron donor (65). However, genomic investigations of 26 *Schinkia* genomes did not detect *frdAB* genes encoding the membrane-bound fumarate reductase complex, suggesting previously reported fumarate reduction capacity of *Schinkia* members warrants re-evaluation. Strain CFF1 utilizes fumarate as an electron donor for aerobic (i.e., O_2_) and anaerobic (i.e., NO_3_^−^, N_2_O) respiration. The measured stoichiometry between fumarate consumption and N_2_O reduction supports complete oxidation of fumarate to CO_2_ (Table 2). Strain CFF1 utilizes C4 (fumarate, succinate) and C3 (lactate, pyruvate, propionate) central metabolism carboxylic acids as electron donors, whereas C2 (acetate) and C1 (formate) carboxylic acids, as well as H_2_, are not utilized. This electron donor utilization pattern contrasts canonical denitrifiers, including the model organisms *Paracoccus denitrificans* (71) and *Dechloromonas aromatica* (72, 73), which utilize acetate, formate, or H_2_ as electron donors.

Complete denitrifiers carrying clade I *nosZ* achieve comparable growth yields per mol of electrons transferred to NO_3_^−^ (NO_3_^−^→ N_2_) or to N_2_O as an electron acceptor (~5 mg biomass mmol electrons^−1^, respectively) (74, 75). While the growth yield of 5.08 ± 1.19 mg strain CFF1 biomass mmol electrons^−1^ during N_2_O respiration is comparable to yields of clade I and clade II N_2_O reducers (59, 74, 75), the growth yields with NO_3_^−^ or NO_2_^−^ are about two-fold higher and exceed the measured yield with O_2_ as the electron acceptor (Table 3). Facultative denitrifiers generally have higher growth yields with O_2_ than with NO_3_^−^ as an electron acceptor (74). We observed impaired growth of strain CFF1 at atmospheric concentrations of O_2_ (21.2 kPa), but not at lower O_2_ partial pressures, suggesting that reactive oxygen species may impact growth of strain CFF1.

### Unusual response of strain CFF1 to acetylene

Acetylene is a well-known inhibitor to N_2_O reduction catalyzed by clade I, clade II, and clade III NosZ (14, 76) and has been used to estimate denitrification potential in microcosms as well as mixed and axenic cultures (76, 77). Some soil microcosm studies have reported incomplete inhibition of N_2_O reduction by acetylene, which was attributed to acetylene depletion by resident acetylene-degrading anaerobes (78, 79). Interestingly, N_2_O reduction by strain CFF1 is not inhibited by high acetylene concentrations. Gas chromatographic analysis confirmed the presence of the initial amount of acetylene throughout the incubation, indicating that acetylene consumption or loss cannot explain N_2_O reduction activity in strain CFF1 cultures. Incomplete N_2_O consumption at reduced rates in the presence of acetylene has been observed in axenic cultures of *Thiobacillus denitrificans* strain ACN20 (80), and strain CFF1 represents a novel type of soil isolate whose N_2_O reduction activity completely resists acetylene inhibition. Previous studies have documented that as much as 11.7% of N_2_O produced during soil denitrification was further reduced into N_2_ in the presence of 10% (vol/vol) acetylene, although the N_2_O-reducing taxa potentially tolerant to acetylene were not identified (78, 81). The acetylene inhibition method is often used to gauge a soil’s denitrification potential and denitrification rates (78), and the presence of acetylene-resistant N_2_O-reducing bacteria, such as strain CFF1, would lead to an underestimation of the true soil denitrification activity. Apparently, the responses of N_2_O-reducing bacteria to acetylene differ, and without detailed molecular-level mechanistic understanding of acetylene inhibition, information generated using the acetylene inhibition technique should be interpreted cautiously.

### Adaptation of *Schinkia acidoalkalinis* strain CFF1 to a broad pH range

The genome of *Schinkia acidoalkalinis* strain CFF1 harbors unusually high rRNA gene copy numbers—16 5S, 15 16S, and 15 23S rRNA genes—which substantially exceed those observed in other *Schinkia* species, ranging from 1 to 12 (5S), 1 to 4 (16S), and 1 to 4 (23S) copies (Table S1). The rRNA gene copy numbers in bacterial genomes have been linked to the organisms’ lifestyles, and bacteria with high copy numbers of rRNA genes can more rapidly respond to fluctuating environmental conditions (82, 83). Future research should explore if such traits contribute to strain CFF1’s ability to maintain N_2_O reduction activity in soils across a wide pH range.

Most complete denitrifiers harbor a single clade I *nosZ* gene (12), and only one isolate genome and six MAGs have been reported to carry two *nosZ* variants, which occur juxtapositioned on the respective genomes (24, 84). *Thauera linaloolentis* strain 47Lol, *Dechlorobacter hydrogenophilus* strain LT-1, and *Thauera butanivorans* strain NBRC 103042 are the only isolates harboring both one clade I and one clade II *nosZ* gene (85). In contrast, the four *nosZ* genes are dispersed throughout strain CFF1 genome, each positioned within a set comprising at least three canonical *nos* accessory genes. The genetic redundancy of *nos* gene clusters in strain CFF1 raises intriguing questions regarding regulatory flexibility of this bacterium’s N_2_O-reducing machinery. For example, *Thauera linaloolentis* strain 47Lol harbors a clade I and a clade II *nosZ* gene and downregulates clade II NosZ expression when NO_3_^−^ is present, whereas the expression of clade I NosZ is not impacted (85). The presence of multiple *nos* clusters may reflect regulatory diversification, allowing fine-tuned expression of NosZ in response to specific environmental cues, although experimental verification would be needed.

Global shifts in proteomes have been documented in bacteria exposed to pH stress, where acidic pH was linked to increased abundances of proteins involved in cell cycle–linked morphogenesis and envelope remodeling (86). Beyond *nosZ* expression, the comparative proteomic analyses indicate broader cellular adaptations that support a pH generalist strategy of strain CFF1. Under low pH conditions, higher abundances of proteins involved in energy generation, translation, and homeostatic regulation were observed, pointing to a multifaceted, coordinated response system to sustain growth at pH 4.5 and 8.5. Proteomic analysis of the model denitrifier *Paracoccus denitrificans* demonstrated a stark effect of pH on the expression of clade I NosZ, with substantially higher expression levels observed at pH 7.5 than at pH 6.5 (86). In contrast, the proteomic analyses conducted on strain CFF1 cultures revealed that two of the four *nosZ* variants (i.e., *nosZ*_1 and *nosZ*_3) are consistently expressed when N_2_O was provided as an electron acceptor under both acidic and alkaline cultivation conditions. The lack of differential NosZ abundance under acidic versus alkaline pH suggests that the external pH does not regulate the activity of these *nos* gene clusters (Fig. 4A). Such regulatory stability may confer a fitness advantage in environments with frequent pH fluctuations, allowing strain CFF1 to maintain N_2_O reduction activity regardless of external pH conditions. In contrast, *nosZ*_2 and *nosZ*_4 remained silent under the tested pH regimes, and their expression triggers remain unknown. The *nosZ*_2 cluster lacks *nosL*, but the absence of this gene is an unlikely explanation for the lack of NosZ_2 synthesis because the *nos*_3 gene cluster also lacks *nosL,* but NosZ_3 was detected. Of note, the proteomic detection of diagnostic peptides does not prove that a catalytically active enzyme is synthesized because a non-functional apoprotein would give rise to the same peptides. *nos*_4 lacks a *nosB* gene, and a *Wolinella succinogenes* mutant with a defective *nosB* lost N_2_O reduction activity (87), suggesting that the *nos*_4 gene cluster in strain CFF1 may not be functional.

### Environmental and biotechnological implications

N_2_O formation in soils is highly dynamic over spatial and temporal scales (88), with rainfall, fertilization, and freeze-thaw demonstrated to cause “hot spots and hot moments” of exceptionally high soil N_2_O formation (89, 90). Strain CFF1 has an r-strategist lifestyle and uses a variety of electron donors, exhibits short lag phases and fast growth rates (maximal µ of 0.13 h^−1^ with N_2_O as an electron acceptor), and sporulates to survive famine, suggesting that this bacterium is well-adapted to contribute to N_2_O consumption in dynamic soil environments with fluctuating pH conditions. Field trials delivering N_2_O-reducing bacteria in organic wastes, such as digestate, reduced farmland N_2_O emissions by 50–95% (21). The bioaugmentation agent, *Cloacibacterium* sp. strain CB-01, showed strong and persistent N_2_O reduction in circumneutral soils, but its activity waned in acidic soils (34). *Schinkia acidoalkalinis* strain CFF1 maintains robust N_2_O reduction across a broad pH range, a desirable feature for stimulation of N_2_O reduction activity in soils with spatial and temporal pH heterogeneity (30, 91). As a complete denitrifier, strain CFF1 has the potential to contribute to N_2_O formation when NO_3_^−^ or NO_2_^−^ are available; however, this feature does not preclude its utility as a bioaugmentation agent. For example, the ability to grow with NO_3_^−^ or NO_2_^−^ as electron acceptors may increase the longevity of the bioaugmentation agent and establish robust *in situ* N_2_O consumption at rates that exceed local N_2_O production rates. This concept is illustrated by several *Bradyrhizobium* strains that transiently generate N_2_O, yet still reduce net field N_2_O emissions because N_2_O reduction surpasses N_2_O production (92, 93). While strain CFF1 holds potential in mitigating N_2_O emissions, further investigation is needed to evaluate its performance in soil ecosystems. Specifically, its ability to establish, persist, and compete with native microbial communities under *in situ* conditions warrants evaluation.

### Etymology of *Schinkia acidoalkalinis* sp. nov

*Schinkia acidoalkalinis* (schin’ki.a a.ci.do.al.ka.li’nis. N.L. fem. n. *Schinkia*, named after Bernhard Schink, German microbiologist known for his contributions to anaerobic microbiology; N.L. adj. *acidoalkalinis*, composed of L. *acidus*, sour, acidic, and N.L. *alkalinis*, alkaline; N.L. adj. *acidoalkalinis*, referring to the bacterium’s ability to grow and reduce N_2_O under both acidic and alkaline pH conditions).

## Data Availability

The Illumina short-read and PacBio data sets and the closed genome of *Schinkia acidoakalinis* strain CFF1 are deposited under NCBI BioProject ID PRJNA1221298. The mass spectrometry proteomics raw data and sequence files are available at the ProteomeXchange Consortium via the MassIVE database with the unique data set identifier MSV000099934.

## SUPPLEMENTAL MATERIAL

Supplemental material aem.00069-26-s0001.pdf Methods S1 to S6; Fig. S1 to S7.

Supplemental tables aem.00069-26-s0002.xlsx Tables S1 to S5.

ASM does not own the copyrights to Supplemental Material that may be linked to, or accessed through, an article. The authors have granted ASM a non-exclusive, world-wide license to publish the Supplemental Material files. Please contact the corresponding author directly for reuse.

## References

[1] Zhu G, Shi H, Zhong L, He G, Wang B, Shan J, Han P, Liu T, Wang S, Liu C, et al.. 2025. Nitrous oxide sources, mechanisms and mitigation. Nat Rev Earth Environ 6:574–592. doi:10.1038/s43017-025-00707-5

[2] Tian H, Pan N, Thompson RL, Canadell JG, Suntharalingam P, Regnier P, Davidson EA, Prather M, Ciais P, Muntean M, et al.. 1980. Global nitrous oxide budget (1980–2020). Earth Syst Sci Data 16:2543–2604. doi:10.5194/essd-16-2543-2024

[3] Yin Yongchao, Kara-Murdoch F, Murdoch RW, Yan J, Chen G, Xie Y, Sun Y, Löffler FE. 2024. Nitrous oxide inhibition of methanogenesis represents an underappreciated greenhouse gas emission feedback. ISME J 18:wrae027. doi:10.1093/ismejo/wrae027 38447133 PMC10960958

[4] Yin Y, Yan J, Chen G, Murdoch FK, Pfisterer N, Löffler FE. 2019. Nitrous oxide is a potent inhibitor of bacterial reductive dechlorination. Environ Sci Technol 53:692–701. doi:10.1021/acs.est.8b05871 30558413 PMC6944068

[5] Sullivan MJ, Gates AJ, Appia-Ayme C, Rowley G, Richardson DJ. 2013. Copper control of bacterial nitrous oxide emission and its impact on vitamin B12-dependent metabolism. Proc Natl Acad Sci USA 110:19926–19931. doi:10.1073/pnas.1314529110 24248380 PMC3856849

[6] Zhang L, Yin Y, Sun Y, Liang X, Graham DE, Pierce EM, Löffler FE, Gu B. 2023. Inhibition of methylmercury and methane formation by nitrous oxide in arctic tundra soil microcosms. Environ Sci Technol 57:5655–5665. doi:10.1021/acs.est.2c09457 36976621 PMC10100821

[7] Wasson PA, McRose DL. 2026. Nitrous oxide produced by denitrifying pseudomonads inhibits the growth of rhizosphere bacteria by inactivating the cobalamin-dependent methionine synthase. mBio 17:e0269925. doi:10.1128/mbio.02699-25 41778806 PMC13059711

[8] Smith C, Hill AK, Torrente-Murciano L. 2020. Current and future role of Haber–Bosch ammonia in a carbon-free energy landscape. Energy Environ Sci 13:331–344. doi:10.1039/C9EE02873K

[9] Davidson EA. 2009. The contribution of manure and fertilizer nitrogen to atmospheric nitrous oxide since 1860. Nature Geosci 2:659–662. doi:10.1038/ngeo608

[10] Kuypers MMM, Marchant HK, Kartal B. 2018. The microbial nitrogen-cycling network. Nat Rev Microbiol 16:263–276. doi:10.1038/nrmicro.2018.9 29398704

[11] Leon-Palmero E, Morales-Baquero R, Thamdrup B, Löscher C, Reche I. 2025. Sunlight drives the abiotic formation of nitrous oxide in fresh and marine waters. Science 387:1198–1203. doi:10.1126/science.adq0302 40080575

[12] Sanford RA, Wagner DD, Wu Q, Chee-Sanford JC, Thomas SH, Cruz-García C, Rodríguez G, Massol-Deyá A, Krishnani KK, Ritalahti KM, Nissen S, Konstantinidis KT, Löffler FE. 2012. Unexpected nondenitrifier nitrous oxide reductase gene diversity and abundance in soils. Proc Natl Acad Sci USA 109:19709–19714. doi:10.1073/pnas.1211238109 23150571 PMC3511753

[13] Li G, Hong H, Lin W, Ji Q. 2024. Substrate competition of diazotrophic nitrous oxide assimilation over dinitrogen fixation. JGR Biogeosciences 129:e2024JG008187. doi:10.1029/2024JG008187

[14] He G, Wang W, Chen G, Xie Y, Parks JM, Davin ME, Hettich RL, Konstantinidis KT, Löffler FE. 2025. A novel bacterial protein family that catalyses nitrous oxide reduction. Nature 646:152–160. doi:10.1038/s41586-025-09401-4 40836093

[15] Shan J, Sanford RA, Chee-Sanford J, Ooi SK, Löffler FE, Konstantinidis KT, Yang WH. 2021. Beyond denitrification: the role of microbial diversity in controlling nitrous oxide reduction and soil nitrous oxide emissions. Glob Chang Biol 27:2669–2683. doi:10.1111/gcb.15545 33547715

[16] Liu B, Frostegård Å, Bakken LR. 2014. Impaired reduction of N_2_O to N_2_ in acid soils is due to a posttranscriptional interference with the expression of nosZ. mBio 5:e01383-14. doi:10.1128/mBio.01383-14 24961695 PMC4073493

[17] Bergaust L, Mao Y, Bakken LR, Frostegård A. 2010. Denitrification response patterns during the transition to anoxic respiration and posttranscriptional effects of suboptimal pH on nitrous [corrected] oxide reductase in Paracoccus denitrificans. Appl Environ Microbiol 76:6387–6396. doi:10.1128/AEM.00608-10 20709842 PMC2950438

[18] Frostegård Å, Vick SHW, Lim NYN, Bakken LR, Shapleigh JP. 2022. Linking meta-omics to the kinetics of denitrification intermediates reveals pH-dependent causes of N_2_O emissions and nitrite accumulation in soil. ISME J 16:26–37. doi:10.1038/s41396-021-01045-2 34211102 PMC8692524

[19] Lawson CE, Harcombe WR, Hatzenpichler R, Lindemann SR, Löffler FE, O’Malley MA, García Martín H, Pfleger BF, Raskin L, Venturelli OS, Weissbrodt DG, Noguera DR, McMahon KD. 2019. Common principles and best practices for engineering microbiomes. Nat Rev Microbiol 17:725–741. doi:10.1038/s41579-019-0255-9 31548653 PMC8323346

[20] Lutz S, Bodenhausen N, Hess J, Valzano-Held A, Waelchli J, Deslandes-Hérold G, Schlaeppi K, van der Heijden MGA. 2023. Soil microbiome indicators can predict crop growth response to large-scale inoculation with arbuscular mycorrhizal fungi. Nat Microbiol 8:2277–2289. doi:10.1038/s41564-023-01520-w 38030903 PMC10730404

[21] Hiis EG, Vick SHW, Molstad L, Røsdal K, Jonassen KR, Winiwarter W, Bakken LR. 2024. Unlocking bacterial potential to reduce farmland N_2_O emissions. Nature 630:421–428. doi:10.1038/s41586-024-07464-3 38811724 PMC11168931

[22] He G, Löffler FE. 2024. Nitrogen-hungry bacteria added to farm soil curb greenhouse-gas emissions. Nature 630:310–311. doi:10.1038/d41586-024-01363-3 38858486

[23] Kaminsky LM, Trexler RV, Malik RJ, Hockett KL, Bell TH. 2019. The inherent conflicts in developing soil microbial inoculants. Trends Biotechnol 37:140–151. doi:10.1016/j.tibtech.2018.11.011 30587413

[24] Jones CM, Welsh A, Throbäck IN, Dörsch P, Bakken LR, Hallin S. 2011. Phenotypic and genotypic heterogeneity among closely related soil-borne N_2_ - and N_2_O-producing Bacillus isolates harboring the nosZ gene. FEMS Microbiol Ecol 76:541–552. doi:10.1111/j.1574-6941.2011.01071.x 21348884

[25] Bueno E, Mania D, Frostegard Ǻsa, Bedmar EJ, Bakken LR, Delgado MJ. 2015. Anoxic growth of Ensifer meliloti 1021 by N_2_O-reduction, a potential mitigation strategy. Front Microbiol 6:537. doi:10.3389/fmicb.2015.00537 26074913 PMC4443521

[26] Kim H, Park D, Yoon S. 2017. pH control enables simultaneous enhancement of nitrogen retention and N_2_O reduction in shewanella loihica Strain PV-4. Front Microbiol 8. doi:10.3389/fmicb.2017.01820 PMC5611402 28979255

[27] He G, Chen G, Xie Y, Swift CM, Ramirez D, Cha G, Konstantinidis KT, Radosevich M, Löffler FE. 2024. Sustained bacterial N_2_O reduction at acidic pH. Nat Commun 15:4092. doi:10.1038/s41467-024-48236-x 38750010 PMC11096178

[28] Awala SI, Gwak JH, Kim Y, Jung MY, Dunfield PF, Wagner M, Rhee SK. 2024. Nitrous oxide respiration in acidophilic methanotrophs. Nat Commun 15:4226. doi:10.1038/s41467-024-48161-z 38762502 PMC11102522

[29] Wang X, Meng L, Li H, Zhang Y, Li M, Xiang B, Zhu J, Li C, Yang C, Yu S, Pang X, Wei J, Zhang M, Bakken LR, Frostegård Å, Zhang L, Zhang X. 2025. Two small proteins enable N_2_O reduction in acidic environments. bioRxiv. doi:10.64898/2025.12.16.694761

[30] Zhu K, Ye X, Ran H, Zhang P, Wang G. 2022. Contrasting effects of straw and biochar on microscale heterogeneity of soil O_2_ and pH: implication for N2O emissions. Soil Biology and Biochemistry 166:108564. doi:10.1016/j.soilbio.2022.108564

[31] Sorokin DY, Kuenen JG, Jetten MS. 2001. Denitrification at extremely high pH values by the alkaliphilic, obligately chemolithoautotrophic, sulfur-oxidizing bacterium Thioalkalivibrio denitrificans strain ALJD. Arch Microbiol 175:94–101. doi:10.1007/s002030000210 11285746

[32] Zhou Y, Zhao S, Suenaga T, Kuroiwa M, Riya S, Terada A. 2022. Nitrous oxide-sink capability of denitrifying bacteria impacted by nitrite and pH. Chem Eng J 428:132402. doi:10.1016/j.cej.2021.132402

[33] Bueno E, Mania D, Mesa S, Bedmar EJ, Frostegård Å, Bakken LR, Delgado MJ. 2022. Regulation of the emissions of the greenhouse gas nitrous oxide by the soybean endosymbiont Bradyrhizobium diazoefficiens Int J Mol Sci 23:1486. doi:10.3390/ijms23031486 35163408 PMC8836242

[34] Jonassen KR, Ormåsen I, Duffner C, Hvidsten TR, Bakken LR, Vick SHW. 2022. A dual enrichment strategy provides soil- and digestate-competent nitrous oxide-respiring bacteria for mitigating climate forcing in agriculture. mBio 13:e0078822. doi:10.1128/mbio.00788-22 35638872 PMC9239227

[35] Fukushi M, Mino S, Tanaka H, Nakagawa S, Takai K, Sawabe T. 2020. Biogeochemical implications of N2O-reducing thermophilic Campylobacteria in deep-sea vent fields, and the description of Nitratiruptor labii sp. nov. iScience 23:101462. doi:10.1016/j.isci.2020.101462 32866828 PMC7476070

[36] Lycus P, Lovise Bøthun K, Bergaust L, Peele Shapleigh J, Reier Bakken L, Frostegård Å. 2017. Phenotypic and genotypic richness of denitrifiers revealed by a novel isolation strategy. ISME J 11:2219–2232. doi:10.1038/ismej.2017.82 28696424 PMC5607364

[37] Brinton W, Basso B, Millar N, Covey K, Bettigole C, Jagadamma S, Loeffler F, Kolodney S. 2025. An inter‐laboratory comparison of soil organic carbon analysis on a farm with four agricultural management systems. Agron J 117:e270018. doi:10.1002/agj2.70018

[38] Chen G, Jiang N, Villalobos Solis MI, Kara Murdoch F, Murdoch RW, Xie Y, Swift CM, Hettich RL, Löffler FE. 2021. Anaerobic microbial metabolism of dichloroacetate. mBio 12:e00537-21. doi:10.1128/mBio.00537-21 33906923 PMC8092247

[39] Di Tommaso P, Chatzou M, Floden EW, Barja PP, Palumbo E, Notredame C. 2017. Nextflow enables reproducible computational workflows. Nat Biotechnol 35:316–319. doi:10.1038/nbt.3820 28398311

[40] Löffler FE, Sun Q, Li J, Tiedje JM. 2000. 16S rRNA gene-based detection of tetrachloroethene-dechlorinating Desulfuromonas and Dehalococcoides species. Appl Environ Microbiol 66:1369–1374. doi:10.1128/AEM.66.4.1369-1374.2000 10742213 PMC91994

[41] Krakau S, Straub D, Gourlé H, Gabernet G, Nahnsen S. 2022. Nf-core/mag: a best-practice pipeline for metagenome hybrid assembly and binning. NAR Genom Bioinform 4:lqac007. doi:10.1093/nargab/lqac007 35118380 PMC8808542

[42] Koren S, Walenz BP, Berlin K, Miller JR, Bergman NH, Phillippy AM. 2017. Canu: scalable and accurate long-read assembly via adaptive k-mer weighting and repeat separation. Genome Res 27:722–736. doi:10.1101/gr.215087.116 28298431 PMC5411767

[43] Kolmogorov M, Yuan J, Lin Y, Pevzner PA. 2019. Assembly of long, error-prone reads using repeat graphs. Nat Biotechnol 37:540–546. doi:10.1038/s41587-019-0072-8 30936562

[44] Hunt M, Silva ND, Otto TD, Parkhill J, Keane JA, Harris SR. 2015. Circlator: automated circularization of genome assemblies using long sequencing reads. Genome Biol 16:294. doi:10.1186/s13059-015-0849-0 26714481 PMC4699355

[45] Wick RR, Holt KE. 2022. Polypolish: short-read polishing of long-read bacterial genome assemblies. PLoS Comput Biol 18:e1009802. doi:10.1371/journal.pcbi.1009802 35073327 PMC8812927

[46] Jain C, Rodriguez-R LM, Phillippy AM, Konstantinidis KT, Aluru S. 2018. High throughput ANI analysis of 90K prokaryotic genomes reveals clear species boundaries. Nat Commun 9:5114. doi:10.1038/s41467-018-07641-9 30504855 PMC6269478

[47] Grant JR, Enns E, Marinier E, Mandal A, Herman EK, Chen C-Y, Graham M, Van Domselaar G, Stothard P. 2023. Proksee: in-depth characterization and visualization of bacterial genomes. Nucleic Acids Res 51:W484–W492. doi:10.1093/nar/gkad326 37140037 PMC10320063

[48] Vernikos GS, Parkhill J. 2006. Interpolated variable order motifs for identification of horizontally acquired DNA: revisiting the Salmonella pathogenicity islands. Bioinformatics 22:2196–2203. doi:10.1093/bioinformatics/btl369 16837528

[49] Brown CL, Mullet J, Hindi F, Stoll JE, Gupta S, Choi M, Keenum I, Vikesland P, Pruden A, Zhang L. 2022. mobileOG-db: a manually curated database of protein families mediating the life cycle of bacterial mobile genetic elements. Appl Environ Microbiol 88:e0099122. doi:10.1128/aem.00991-22 36036594 PMC9499024

[50] Wishart DS, Han S, Saha S, Oler E, Peters H, Grant JR, Stothard P, Gautam V. 2023. PHASTEST: faster than PHASTER, better than PHAST. Nucleic Acids Res 51:W443–W450. doi:10.1093/nar/gkad382 37194694 PMC10320120

[51] Cantalapiedra CP, Hernández-Plaza A, Letunic I, Bork P, Huerta-Cepas J. 2021. eggNOG-mapper v2: functional annotation, orthology assignments, and domain prediction at the metagenomic scale. Mol Biol Evol 38:5825–5829. doi:10.1093/molbev/msab293 34597405 PMC8662613

[52] Katoh K, Standley DM. 2013. MAFFT multiple sequence alignment software version 7: improvements in performance and usability. Mol Biol Evol 30:772–780. doi:10.1093/molbev/mst010 23329690 PMC3603318

[53] Capella-Gutiérrez S, Silla-Martínez JM, Gabaldón T. 2009. trimAl: a tool for automated alignment trimming in large-scale phylogenetic analyses. Bioinformatics 25:1972–1973. doi:10.1093/bioinformatics/btp348 19505945 PMC2712344

[54] Kozlov AM, Darriba D, Flouri T, Morel B, Stamatakis A. 2019. RAxML-NG: a fast, scalable and user-friendly tool for maximum likelihood phylogenetic inference. Bioinformatics 35:4453–4455. doi:10.1093/bioinformatics/btz305 31070718 PMC6821337

[55] Letunic I, Bork P. 2024. Interactive Tree of Life (iTOL) v6: recent updates to the phylogenetic tree display and annotation tool. Nucleic Acids Res 52:W78–W82. doi:10.1093/nar/gkae268 38613393 PMC11223838

[56] Välikangas T, Suomi T, Elo LL. 2016. A systematic evaluation of normalization methods in quantitative label-free proteomics. Brief Bioinform 19:bbw095. doi:10.1093/bib/bbw095 PMC5862339 27694351

[57] Rohart F, Gautier B, Singh A, Lê Cao K-A. 2017. mixOmics: an R package for ‘omics feature selection and multiple data integration. PLoS Comput Biol 13:e1005752. doi:10.1371/journal.pcbi.1005752 29099853 PMC5687754

[58] Ritchie ME, Phipson B, Wu D, Hu Y, Law CW, Shi W, Smyth GK. 2015. Limma powers differential expression analyses for RNA-sequencing and microarray studies. Nucleic Acids Res 43:e47–e47. doi:10.1093/nar/gkv007 25605792 PMC4402510

[59] Yoon S, Nissen S, Park D, Sanford RA, Löffler FE. 2016. Nitrous oxide reduction kinetics distinguish bacteria harboring clade I NosZ from those harboring Clade II NosZ. Appl Environ Microbiol 82:3793–3800. doi:10.1128/AEM.00409-16 27084012 PMC4907195

[60] Sander R. 2015. Compilation of Henry’s law constants (version 4.0) for water as solvent. Atmos Chem Phys 15:4399–4981. doi:10.5194/acp-15-4399-2015

[61] Gupta RS, Patel S, Saini N, Chen S. 2020. Robust demarcation of 17 distinct Bacillus species clades, proposed as novel Bacillaceae genera, by phylogenomics and comparative genomic analyses: description of Robertmurraya kyonggiensis sp. nov. and proposal for an emended genus Bacillus limiting it only to the members of the Subtilis and Cereus clades of species. Int J Syst Evol Microbiol 70:5753–5798. doi:10.1099/ijsem.0.004475 33112222

[62] Rodriguez-R LM, Conrad RE, Viver T, Feistel DJ, Lindner BG, Venter SN, Orellana LH, Amann R, Rossello-Mora R, Konstantinidis KT. 2024. An ANI gap within bacterial species that advances the definitions of intra-species units. mBio 15:e0269623. doi:10.1128/mbio.02696-23 38085031 PMC10790751

[63] Goris J, Konstantinidis KT, Klappenbach JA, Coenye T, Vandamme P, Tiedje JM. 2007. DNA-DNA hybridization values and their relationship to whole-genome sequence similarities. Int J Syst Evol Microbiol 57:81–91. doi:10.1099/ijs.0.64483-0 17220447

[64] Müller C, Zhang L, Zipfel S, Topitsch A, Lutz M, Eckert J, Prasser B, Chami M, Lü W, Du J, Einsle O. 2022. Molecular interplay of an assembly machinery for nitrous oxide reductase. Nature 608:626–631. doi:10.1038/s41586-022-05015-2 35896743

[65] Pichinoty F, De Barjac H, Mandel M, Asselineau J. 1983. Description of Bacillus azotoformans sp. nov. Int J Syst Bacteriol 33:660–662. doi:10.1099/00207713-33-3-660

[66] Bao P, Xiao K-Q, Wang H-J, Xu H, Xu P-P, Jia Y, Häggblom MM, Zhu Y-G. 2016. Characterization and Potential applications of a selenium nanoparticle producing and nitrate reducing bacterium Bacillus oryziterrae sp. nov. Sci Rep 6:34054. doi:10.1038/srep34054 27677458 PMC5039721

[67] Barnum TP, Crits-Christoph A, Molla M, Carini P, Lee HH, Ostrov N. 2024. Predicting microbial growth conditions from amino acid composition. Microbiology. doi:10.1101/2024.03.22.586313

[68] Shi W, Li Y, Zhang W. 2024. Screening and functional characterization of isocitrate lyase AceA in the biofilm formation of Vibrio alginolyticus. Appl Environ Microbiol 90:e0069724. doi:10.1128/aem.00697-24 39377591 PMC11577800

[69] Hein S, Simon J. 2019. Bacterial nitrous oxide respiration: electron transport chains and copper transfer reactions. Edited by R. K. Poole. Adv Microb Physiol 75:137–175. doi:10.1016/bs.ampbs.2019.07.001 31655736

[70] Qiu Y, Zhang Y, Zhang K, Xu X, Zhao Y, Bai T, Zhao Y, Wang H, Sheng X, Bloszies S, et al.. 2024. Intermediate soil acidification induces highest nitrous oxide emissions. Nat Commun 15:2695. doi:10.1038/s41467-024-46931-3 38538640 PMC10973416

[71] Zhou J, Deng W, Wu J, Xiang H, Shen X, Lin J-G, Hong Y. 2024. Respiration and growth of Paracoccus denitrificans R-1 with nitrous oxide as an electron acceptor. Microbiol Spectr 12:e0381123. doi:10.1128/spectrum.03811-23 38647341 PMC11237620

[72] Coates JD, Chakraborty R, Lack JG, O’Connor SM, Cole KA, Bender KS, Achenbach LA. 2001. Anaerobic benzene oxidation coupled to nitrate reduction in pure culture by two strains of Dechloromonas. Nature 411:1039–1043. doi:10.1038/35082545 11429602

[73] Duffner C, Kublik S, Fösel B, Frostegård Å, Schloter M, Bakken L, Schulz S. 2022. Genotypic and phenotypic characterization of hydrogenotrophic denitrifiers. Environ Microbiol. doi:10.1111/1462-2920.15921:1887-1901 35106904

[74] Gao Y, Mania D, Mousavi SA, Lycus P, Arntzen MØ, Woliy K, Lindström K, Shapleigh JP, Bakken LR, Frostegård Å. 2021. Competition for electrons favours N_2_O reduction in denitrifying Bradyrhizobium isolates. Environ Microbiol 23:2244–2259. doi:10.1111/1462-2920.15404 33463871

[75] Koike I, Hattori A. 1975. Energy yield of denitrification: an estimate from growth yield in continuous cultures of Pseudomonas denitrificans under nitrate-, nitrite- and nitrous oxide-limited conditions. J Gen Microbiol 88:11–19. doi:10.1099/00221287-88-1-11 1151328

[76] Yoon S, Heo H, Han H, Song D-U, Bakken LR, Frostegård Å, Yoon S. 2023. Suggested role of NosZ in preventing N_2_O inhibition of dissimilatory nitrite reduction to ammonium. mBio 14:e0154023. doi:10.1128/mbio.01540-23 37737639 PMC10653820

[77] Mania D, Woliy K, Degefu T, Frostegård åsa. 2020. A common mechanism for efficient N_2_O reduction in diverse isolates of nodule‐forming bradyrhizobia . Environ Microbiol 22:17–31. doi:10.1111/1462-2920.14731 31271499

[78] Qin S, Hu C, Oenema O. 2012. Quantifying the underestimation of soil denitrification potential as determined by the acetylene inhibition method. Soil Biology and Biochemistry 47:14–17. doi:10.1016/j.soilbio.2011.12.019

[79] Akob DM, Baesman SM, Sutton JM, Fierst JL, Mumford AC, Shrestha Y, Poret-Peterson AT, Bennett S, Dunlap DS, Haase KB, Oremland RS. 2017. Detection of diazotrophy in the acetylene-fermenting anaerobe Pelobacter sp. strain SFB93. Appl Environ Microbiol 83:e01198-17. doi:10.1128/AEM.01198-17 28667109 PMC5561301

[80] Dalsgaard T, Bak F. 1992. Effect of acetylene on nitrous oxide reduction and sulfide oxidation in batch and gradient cultures of Thiobacillus denitrificans. Appl Environ Microbiol 58:1601–1608. doi:10.1128/aem.58.5.1601-1608.1992 1352443 PMC195646

[81] Qin S, Yuan H, Dong W, Hu C, Oenema O, Zhang Y. 2013. Relationship between soil properties and the bias of N_2_O reduction by acetylene inhibition technique for analyzing soil denitrification potential. Soil Biology and Biochemistry 66:182–187. doi:10.1016/j.soilbio.2013.07.016

[82] Klappenbach JA, Dunbar JM, Schmidt TM. 2000. rRNA operon copy number reflects ecological strategies of bacteria. Appl Environ Microbiol 66:1328–1333. doi:10.1128/AEM.66.4.1328-1333.2000 10742207 PMC91988

[83] Condon C, Liveris D, Squires C, Schwartz I, Squires CL. 1995. rRNA operon multiplicity in Escherichia coli and the physiological implications of rrn inactivation. J Bacteriol 177:4152–4156. doi:10.1128/jb.177.14.4152-4156.1995 7608093 PMC177152

[84] Jiang Q, Cao L, Han Y, Li S, Zhao R, Zhang X, Ruff SE, Zhao Z, Peng J, Liao J, Zhu B, Wang M, Lin X, Dong X. 2025. Cold seeps are potential hotspots of deep-sea nitrogen loss driven by microorganisms across 21 phyla. Nat Commun 16:1646. doi:10.1038/s41467-025-56774-1 39952920 PMC11828985

[85] Semedo M, Wittorf L, Hallin S, Song B. 2020. Differential expression of clade I and II N_2_O reductase genes in denitrifying Thauera linaloolentis 47LolT under different nitrogen conditions. FEMS Microbiol Lett 367:fnaa205. doi:10.1093/femsle/fnaa205 33296469

[86] Olaya-Abril A, Hidalgo-Carrillo J, Luque-Almagro VM, Fuentes-Almagro C, Urbano FJ, Moreno-Vivián C, Richardson DJ, Roldán MD. 2021. Effect of pH on the denitrification proteome of the soil bacterium Paracoccus denitrificans PD1222. Sci Rep 11:17276. doi:10.1038/s41598-021-96559-2 34446760 PMC8390676

[87] Hein S, Witt S, Simon J. 2017. Clade II nitrous oxide respiration of Wolinella succinogenes depends on the NosG, -C1, -C2, -H electron transport module, NosB and a Rieske/cytochrome bc complex. Environ Microbiol 19:4913–4925. doi:10.1111/1462-2920.13935 28925551

[88] Shcherbak I, Robertson GP. 2019. Nitrous Oxide (N_2_O) emissions from subsurface soils of agricultural ecosystems. Ecosystems (N Y, Print) 22:1650–1663. doi:10.1007/s10021-019-00363-z

[89] Wagner-Riddle C, Baggs EM, Clough TJ, Fuchs K, Petersen SO. 2020. Mitigation of nitrous oxide emissions in the context of nitrogen loss reduction from agroecosystems: managing hot spots and hot moments. Curr Opin Environ Sustain 47:46–53. doi:10.1016/j.cosust.2020.08.002

[90] Zhang Z, Eddy WC, Stuchiner ER, DeLucia EH, Yang WH. 2024. A conceptual model explaining spatial variation in soil nitrous oxide emissions in agricultural fields. Commun Earth Environ 5:730. doi:10.1038/s43247-024-01875-w

[91] Dong Y, Yang J-L, Zhao X-R, Yang S-H, Mulder J, Dörsch P, Zhang G-L. 2022. Seasonal dynamics of soil pH and N transformation as affected by N fertilization in subtropical China: an in situ 15N labeling study. Science of The Total Environment 816:151596. doi:10.1016/j.scitotenv.2021.151596 34774948

[92] Nishida H, Itakura M, Win KT, Li F, Kakizaki K, Suzuki A, Ohkubo S, Duc LV, Sugawara M, Takahashi K, Shenton M, Masuda S, Shibata A, Shirasu K, Fujisawa Y, Tsubokura M, Akiyama H, Shimoda Y, Minamisawa K, Imaizumi-Anraku H. 2025. Genetic design of soybean hosts and bradyrhizobial endosymbionts reduces N_2_O emissions from soybean rhizosphere. Nat Commun 16:8023. doi:10.1038/s41467-025-63223-6 40908282 PMC12411642

[93] Itakura M, Uchida Y, Akiyama H, Hoshino YT, Shimomura Y, Morimoto S, Tago K, Wang Y, Hayakawa C, Uetake Y, Sánchez C, Eda S, Hayatsu M, Minamisawa K. 2013. Mitigation of nitrous oxide emissions from soils by Bradyrhizobium japonicum inoculation. Nature Clim Change 3:208–212. doi:10.1038/nclimate1734

