# Supplementary material for: A novel Schinkia sp. respires nitrous oxide over a broad pH range
Source: Appl Environ Microbiol. 2026 May 26;92(6):e00069-26. doi: 10.1128/aem.00069-26 (PMC13274427; doi:10.1128/aem.00069-26)
Supplement: Supplemental material — Methods S1 to S6; Fig. S1 to S7. [file aem.00069-26-s0001.pdf]

Supplemental Material

A novel *Schinkia* sp. respires nitrous oxide over a broad pH range

Guang He[1,2‡], Megan E. Davin[3,4], Shaylan Kolodney[5#], Emily Stutzman[5], Asher Wright[5], Robert L. Hettich[4], and Frank E. Löffler[1,2,6*]

[1] Department of Civil and Environmental Engineering, [2] Department of Biosystems Engineering and Soil Science, [3]Bredesen Center for Interdisciplinary Research and Graduate Education, University of Tennessee, Knoxville, Tennessee 37996, USA. [4]Biosciences Division, Oak Ridge National Laboratory, Oak Ridge, Tennessee 37831, USA. [5]Caney Fork Farms, Cartridge, Tennessee, 37030, USA. [6]Department of Biochemistry & Cellular and Molecular Biology, University of Tennessee, Knoxville, Tennessee 37996, USA.

‡ Current address: School of Ecological and Environmental Sciences, East China Normal University, Shanghai, China.

# Current address: Department of Plant Science and Landscape Architecture, University of Maryland, College Park, MD 20742

* Corresponding author: Frank E. Löffler, University of Tennessee, Department of Civil and Environmental Engineering, 325 John D. Tickle Building, 851 Neyland Drive, Knoxville, TN 37996, USA.
Email: frank.loeffler@utk.edu

This PDF file includes:

Supplemental Methods 1-6

Supplemental Figures 1-7

Supplemental References

The Supplemental Tables 1-4 are compiled in a separate Excel file.

Supplemental Methods

Supplemental Methods 1

Preparation of growth medium and implementation of the dual pH enrichment strategy. The basal salt medium consisted of (g L^{-1}): NaCl (1.0); $MgCl_2 \cdot 6H_2O$ (0.5); KH_2PO_4 (7.0); NH_4Cl (0.3); KCl (0.3); $CaCl_2 \cdot 2H_2O$ (0.015); L-cysteine (0.031). The medium also contained 1 mL trace element solution, 1 mL Se/Wo solution, and 0.25 mL resazurin solution (0.1% w/w). The trace element solution contained (mg L^{-1}): $FeCl_2 \cdot 4H_2O$ (1,500); $CoCl_2 \cdot 6H_2O$ (190); $MnCl_2 \cdot 4H_2O$ (100); $ZnCl_2$ (70); H_3BO_3 (6); $Na_2MoO_4 \cdot 2H_2O$ (36); $CuCl_2 \cdot 2H_2O$ (2); and 10 mL HCl (25% solution, w/w). The Se/Wo solution consisted of (mg L^{-1}): $Na_2SeO_3 \cdot 5H_2O$ (6); $NaWO_4 \cdot 2H_2O$ (8), and NaOH (500). The serum bottles with N_2 headspace were sealed with butyl rubber stoppers (Bellco Glass, Vineland, NJ, USA) held in place with aluminum crimps. Following autoclaving, the measured medium pH was ~4.4. Inside a glove box (Coy Laboratory Products, Grass Lake, MI) filled with 97% (v:v) N_2 and 3% H_2, 5 grams of soil was transferred to individual 160-mL glass serum bottles containing 100 mL of medium. The serum bottles were immediately sealed with sterile stoppers and removed from the glove box. Amendments of fumarate (5 mM, 500 µmol) and N_2O (4.16 mM, nominal, 416 µmol) to the cultivation vessels were made using sterile plastic syringes and needles. From one N_2O-reducing microcosm, 3 mL slurry was withdrawn and transferred to fresh pH 4.5 medium amended with N_2O and fumarate. Following N_2O consumption, a 3% (v:v) inoculum was transferred to pH 7 medium, and, following N_2O consumption, a subsequent transfer occurred to pH 4.5 medium. This pH alternating transfer strategy (pH 4.5 → 7 → 4.5, etc.) included six transfers in pH 4.5 medium and six transfers in pH 7 medium (12 transfers total). The pH 7 medium used a lower amount of KH_2PO_4 (0.2 g L^{-1}), and 30 mM sodium bicarbonate was added. The medium pH was adjusted to ~7 with CO_2.

pH range experiments. Different ratios of KH_2PO_4 and K_2HPO_4 (combined concentration of 50 mM) resulted in medium pH values of 4.5, 5.5, and 6.5. The final amounts (L^{-1}) were as follows: pH 4.5, 6.8 g KH_2PO_4; pH 5.5, 6.5 g KH_2PO_4 and 0.3 g K_2HPO_4; pH 6.5, 4.8 g KH_2PO_4 and 2.5 g K_2HPO_4 (1). To achieve pH 3.5, 5 M phosphoric acid was added to the pH 4.5 medium. Medium pH values of 7.5, 8.5, and 9.5 were achieved by titrating 5 M sodium hydroxide to the bicarbonate buffered medium with a lower amount of KH_2PO_4 (0.2 g L^{-1}). All pH values were confirmed in subsamples using a calibrated pH electrode following autoclaving. Culture vessels were incubated at 30ºC.

Temperature and salinity range experiments. The impact of temperature on growth was tested at 4, 10, 20, 30, 40, and 50ºC in standard pH 7 basal salt medium. The impact of NaCl

concentrations on growth was tested by altering the standard pH 7 medium NaCl concentration of 1 g L^{-1} to 2.5, 5, 10, 20 and 30 g L^{-1}.

Supplemental Methods 2

Processing of 16S rRNA amplicon reads. Analysis of amplicon reads was conducted with nf-core/ampliseq v2.3.1 using Nextflow. Software used in nf-core/ampliseq was containerized with Singularity v3.8.6 (2). Amplicon read quality was evaluated with FastQC v0.11.9 (https://www.bioinformatics.babraham.ac.uk/projects/fastqc/) and primer removal used Cutadapt v3.4 (3). Quality control including removal of sequences with poor quality, denoising, and chimera removal was performed, and amplicon sequence variants (ASVs) were inferred using DADA2 (4). Barrnap v0.9 (https://github.com/tseemann/barrnap) was used to discriminate eukaryotic rRNA sequences as potential contamination. ASVs were taxonomically classified based on the Silva v138.1 database (5). Relative and absolute abundances of ASVs were calculated using Qiime2 v2021.8.0 (6).

Supplemental Methods 3

Processing of NovaSeq short reads. Metagenomic short reads, generated from NovaSeq sequencing, were processed using the nf-core/mag pipeline (7) on UT's High Performance and Scientific Computing cluster (Isaac-ng, https://oit.utk.edu/hpsc/isaac-open-enclave-new-kpb/). Individual steps of the nf-core/mag pipeline include evaluation of short read quality with FastQC v0.11.9, followed by quality filtering and Illumina adapter removal using fastp v0.20.1. Short reads mapped to the PhiX genome (GCA_002596845.1, ASM259684v1) with Bowtie2 v2.4.2 were removed. The clean short reads were assembled with Megahit2 v1.2.9 (8). Genome binning of assembled contigs was conducted using MetaBAT2 v2.15 and MaxBin2 v2.2.7 considering read coverage, coverage variance and tetranucleotide frequencies. The output MAGs were refined and dereplicated using DASTool v1.1.6 through a single-copy gene (SCG) scoring strategy coupled to an iterative bin de-replication procedure that produces the highest-scoring set of non-redundant bins (in terms of SCG completeness/contamination) from input bins generated by different binners. The taxonomic placements of the MAGs with at least medium quality were inferred with the GTDB-TK v2.3.2. This workflow produced a fragmented MAG representing strain CFF1, with a completeness of 92.2% and a contamination of 5.23%.
Phylogenomic analysis. Phylogenetic reconstruction included 25 *Schinkia* genomes and the single *Calidifontibacillus* genome (sequences obtained from NCBI, January 2025) and the

complete *Schinkia* sp. strain CFF1 genome (Table S1). The GTDB-TK v2.3.2 toolkit (9) was used to identify, align, and concatenate 120 conserved bacterial marker genes in the 26 *Schinkia* genomes and the *Calidifontibacillus* genome. The ANI of orthologous gene pairs shared between *Schinkia* sp. strain CFF1 and related *Schinkia* (n=25) and *Calidifontibacillus* genomes was computed using fastANI v1.34 (10). The digital DNA-DNA hybridization (dDDH) values were calculated using the web-based Genome-to-Genome Distance Calculator 3.0 (GGDC, https://ggdc.dsmz.de/ggdc.php) (11). The tree annotations of ANI and dDDH data were produced with itol.toolkit v1.1.7 (12) and visualized in the Interactive Tree of Life (iTOL) v6 tool (13).

Genome-based prediction of growth preferences. Growth conditions (oxygen requirement, pH and temperature ranges, salinity preference) of *Schinkia* strains were predicted based on amino acid composition of all protein-coding genes on the respective genomes using GenomeSpot v1.0.1 (14). Preliminary function prediction of coding genes present on *Schinkia* (n=26) and *Calidifontibacillus* (n=1) genomes was performed using Cluster of Orthologous Groups (COG)-emapper tool v2.1.12 (15) and Prokka v1.14.5 (16). The *nar* and *nos* gene clusters encoding NarG and NosZ, respectively, in *Schinkia* and *Calidifontibacillus* genomes were identified with clinker v0.0.31 (17). Graphical representation of *Schinkia* and *Calidifontibacillus* genomes was created using Proksee (18).

Supplemental Methods 4

Quantitative PCR (qPCR). qPCR assay tubes received 10 µL 1X Power SYBR Green, 9.88 µL UltraPure nuclease-free water (Invitrogen, Carlsbad, CA, USA), 300 nM of each primer, and 2 µL template DNA. All qPCR assays were performed using an Applied Biosystems ViiA 7 system (Applied Biosystems, Waltham, MA, USA) with the following amplification conditions: 2 min at 50ºC and 10 min at 95ºC, followed by 40 cycles of 15 s at 95ºC, and 1 min at 60ºC. The qPCR standard curves constructed using serial dilutions of a known copy-number linear DNA fragments had a slope of -3.401, y-intercepts of 34.961, R^2 values of 0.999, and qPCR amplification efficiencies of 96.803%. This standard curve covered a range from 1.1 x 10^3 to 1.1 x 10^{10} target gene copies per assay tube. The genome analysis indicated that the strain CFF1 genome carries fifteen nearly identical 16S rRNA genes (ANI > 99%), and thus the enumeration of cell abundances of strain CFF1 was achieved by dividing the 16S rRNA gene copies by the numbers of 16S rRNA genes on the strain CFF1 genome (i.e., cells = 16S rRNA gene copies / 15).

Supplemental Methods 5

Growth yields determined by dry weight biomass. Strain CFF1 biomass was collected from 90 mL culture suspension samples, washed three times with distilled water in 30-mL centrifuge tubes, and then filtered onto polyvinylidene fluoride (PVDF) membrane filters (0.22 µm, 5 cm diameter, Millipore, Billerica, MA). The membrane filters containing strain CFF1 biomass were placed in pre-weighed individual aluminum cups and dried at 80ºC for 48 hours, after which weight consistency had been achieved. The aluminum cups, each containing a PVDF membrane filter, were then placed in a desiccator to cool to room temperature, and the weight was recorded after weight consistency was observed. The dry biomass weight was calculated by subtracting the pre-measured weight of the respective aluminum cup containing the membrane filter without cells. The growth yield was calculated as mg (dry weight) biomass produced per mol of electrons transferred to the electron acceptor (i.e., O_2, NO_3^-, NO_2^-, or N_2O). Electrons consumed in electron acceptor reduction were calculated based on the following reduction half reactions:

$O_2 + 4e^- + 4H^+ \rightarrow 2H_2O$

$2NO_3^- + 10e^- + 12H^+ \rightarrow N_2 + 6H_2O$

$2NO_2^- + 6e^- + 8H^+ \rightarrow N_2 + 4H_2O$

$N_2O + 2e^- + 2H^+ \rightarrow N_2 + H_2O$

Supplemental Methods 6

Proteomic analysis. Proteomes were extracted using a protein aggregation capture method (19), followed by digestion with a protein to trypsin ratio of 1:75 (w/w). Digested peptides (2 µg per sample) were analyzed using a Vanquish UHPLC system connected to an Orbitrap Q-Exactive Plus mass spectrometer (Thermo Fisher Scientific) utilizing a 1D LC-MS/MS approach. Two µg of peptides was loaded onto a custom analytical column with C18 resin (Kinetex, Phenomenex, Torrance, CA, USA) and desalted for 30 minutes using solvent A (0.1% formic acid in 5% acetonitrile [v/v]) followed by a 185 min gradient ranging from 0-30% solvent B (0.1% formic acid in 70% acetonitrile). A column wash using solvent B was done in between injections. Samples were run in data-dependent acquisition mode. Full-scan MS spectra were collected in the *m/z* 300-1,500 range at a resolution of 70,000 (full-width at half maximum). The twenty most intense precursor ions were selected for MS/MS with an isolation window set to 1.8 *m/z* and a dynamic exclusion window of 30 s. Resulting raw data were searched using Proteome Discoverer (version 2.3.0.523, Thermo Scientific) employing the MS Amanda algorithm (v2.0)

and a target-decoy approach against the corresponding metagenome and a common protein contaminants database (20). Peptide requirements were as follows: fully tryptic peptides, up to two missed cleavage sites, a five-amino acid length minimum, and a false discovery rate of <1%. Peptide quantification was done via area under the curve and with protein quantification calculated by summing unique peptide abundances.

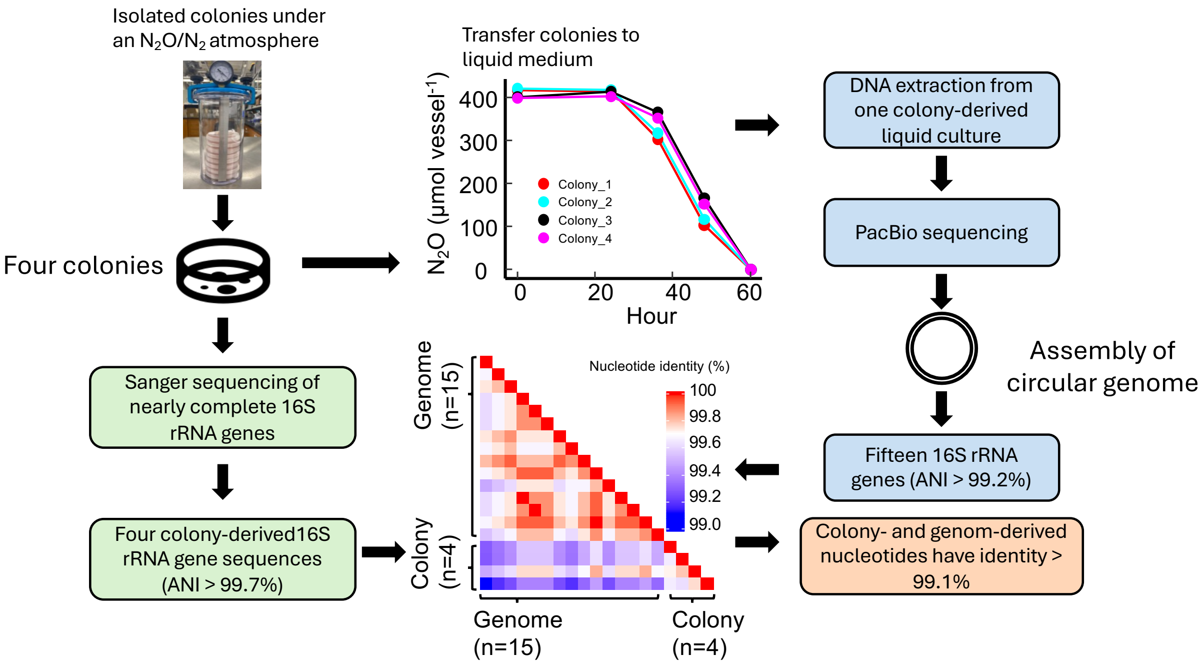

Fig. S1. Schematic workflow to verify purity of strain CFF1. Four nearly complete 16S rRNA gene sequences with >99.7% sequence similarity were derived from the four isolated colonies via Sanger sequencing. Fifteen variant 16S rRNA sequences sharing >99.5% nucleotide identity were derived from the complete genome of strain CFF1. The Illumina short-read and PacBio long-read datasets were generated from the 6th generation enrichment culture and from the pure culture of strain CFF1, respectively. The colony- and genome-derived 16S rRNA sequences exhibited >99.1% nucleotide identity.

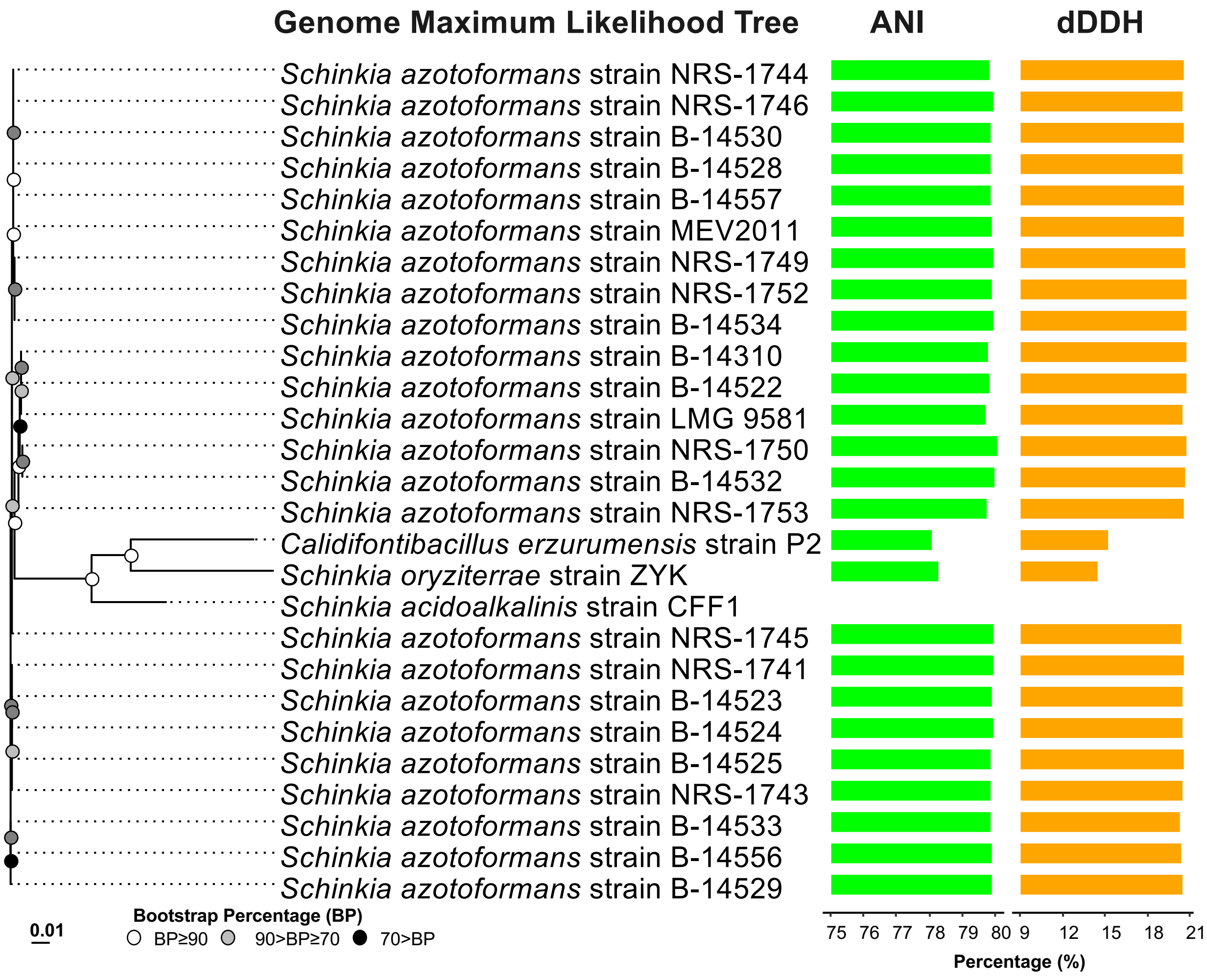

Fig. S2. Phylogenomic, genome-wide average nucleotide identity (ANI), and digital DNA-DNA hybridization (dDDH) analyses of available *Schinkia* (n=26) and *Calidifontibacillus* (n=1) genomes. Phylogenomic analysis used 112 conserved marker genes included the 25 *Schinkia* isolate genomes available in NCBI as of August 2025 and the genome of strain CFF1. The scale bar below the tree topology indicates the nucleotide substitution per site. Bootstrap percentage (BP) values are divided into the categories BP < 70 (solid black circles), 70 ≤ BP < 90 (solid grey circles) and BP ≥ 90 (open circles). The bar plots display the ANI and the dDDH values (%) between *Schinkia acidoalkalinis* strain CFF1 and *Schinkia*/*Calidifontibacillus* isolates with sequenced genomes. The genome accession numbers are summarized in Table S1.

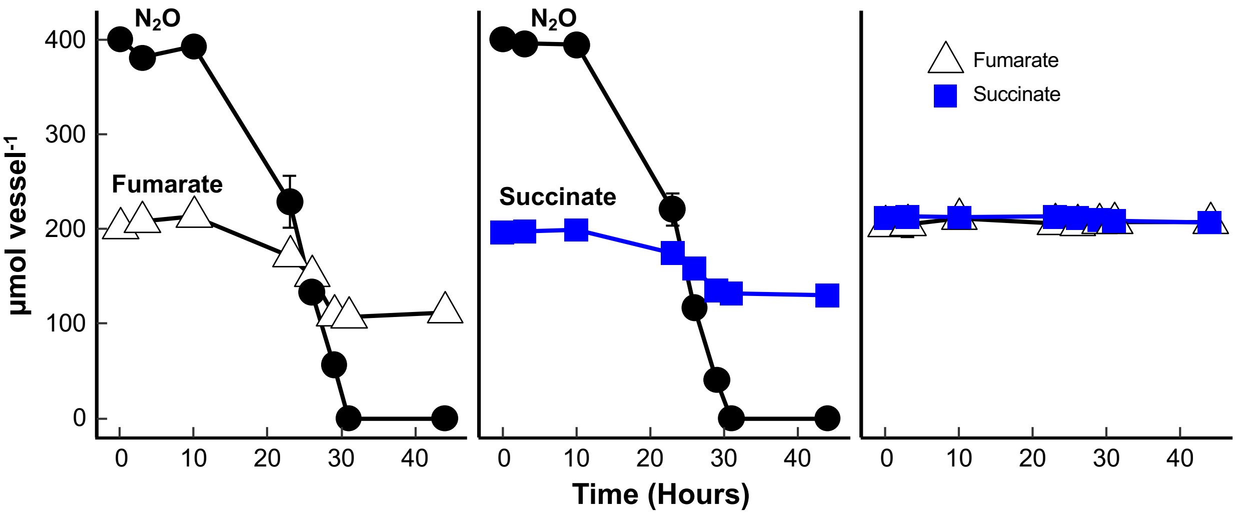

Fig. S3. N_2O reduction by *Schinkia acidoalkalinis* strain CFF1. Consumption of fumarate (open triangles), succinate (blue squares) and N_2O (black circles) in strain CFF1 cultures grown with fumarate (2 mM) plus N_2O (4.16 mM, nominal) (**A**), succinate (2 mM) plus N_2O (**B**), and succinate plus fumarate without N_2O (**C**). Data represent averages and the error bars show the standard deviations of triplicate cultures. No growth and N_2O reduction were observed when electron donors (e.g., fumarate and succinate) were omitted.

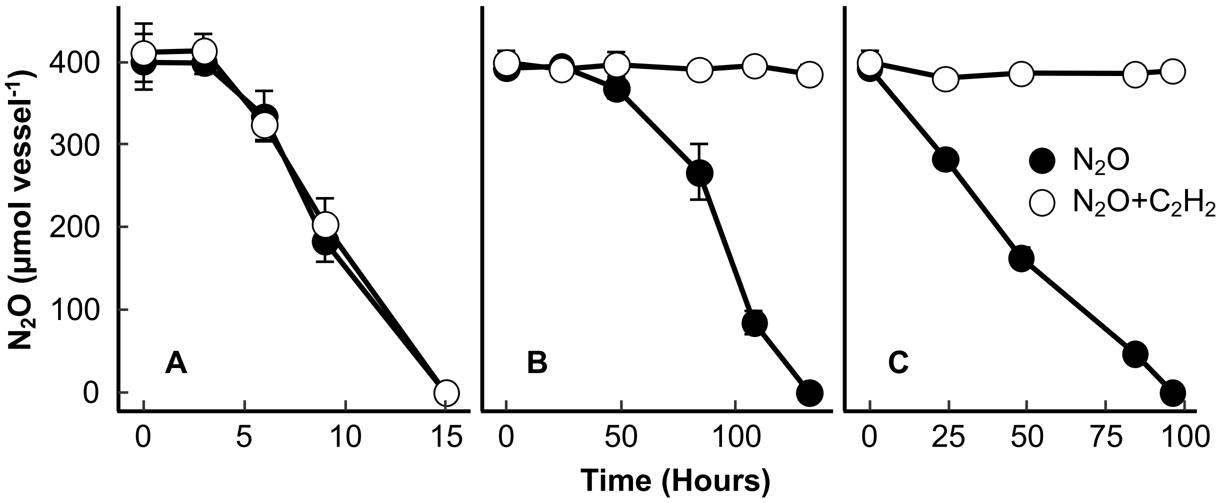

Fig. S4. Impact of acetylene on N_2O reduction in axenic cultures of *Schinkia acidoalkalinis* strain CFF1, *Stutzerimonas stutzeri* strain DCP-Ps1, and *Desulfitobacterium dehalogenans* strain DSM9161. N_2O reduction in cultures of strain CFF1 (**A**), strain DCP-Ps1 (**B**), and strain DSM9161 (**C**) in the presence (open circles) and absence (solid circles) of acetylene (33% of headspace). The cultures were grown in 160 mL serum bottles with 60 mL of headspace. Data represent averages and the error bars show the standard deviations of triplicate cultures.

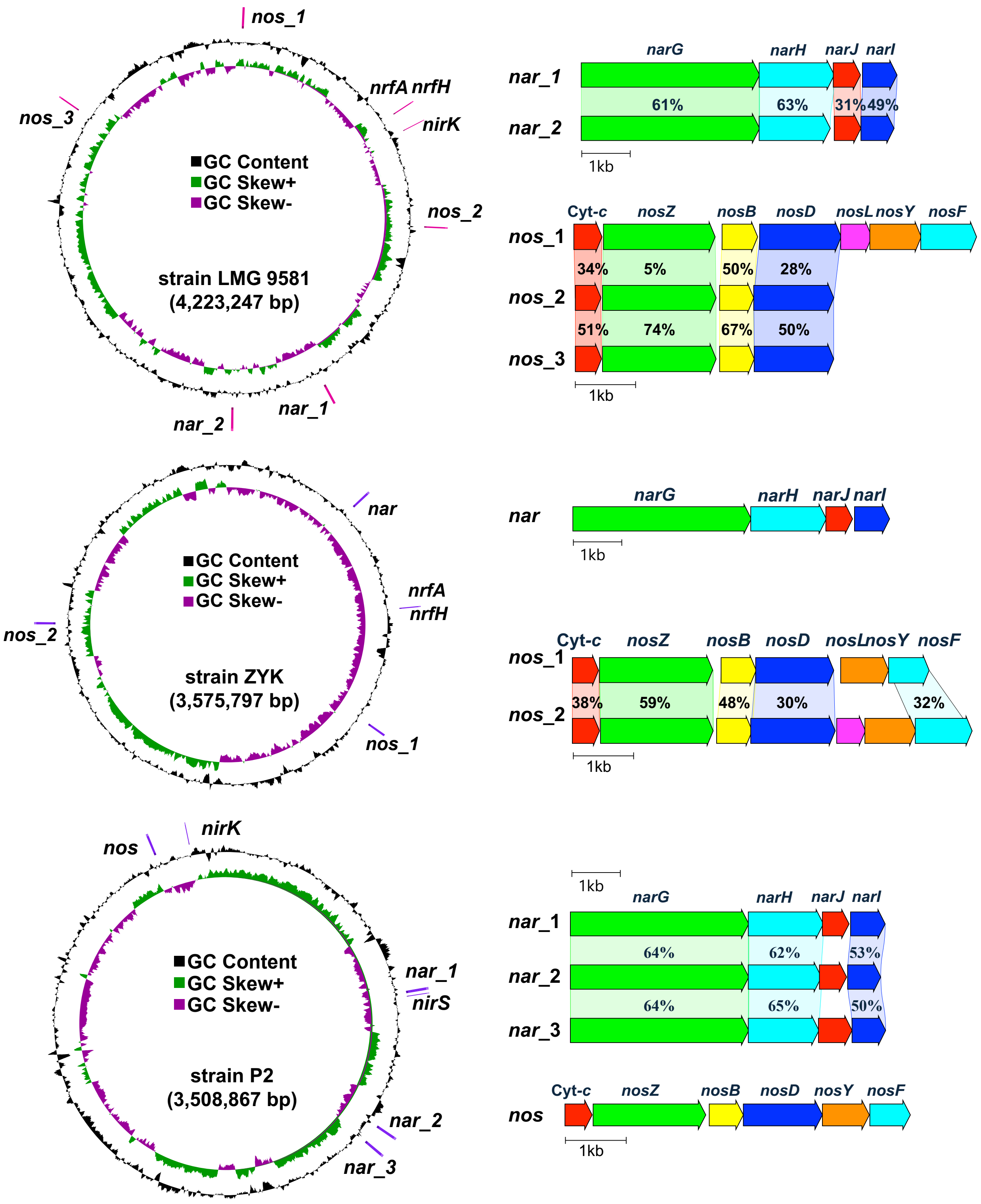

Fig. S5. Comparison of *nar* and *nos* gene clusters and general genomic features in *Schinkia acidoalkalinis* strain CFF1 (Figure 4 in the main text) and three closely related isolates. Circular genome maps (left panels) and *nar* and *nos* gene clusters of *Schinkia azotoformans* strain LMG 9581, *Schinkia oryziterrae* strain ZYK, and *Calidifontibacillus erzurumensis* strain P2. The scale

bars serve as estimators of gene length. The shadings and percentage values between arrows indicate AAI between homologous protein sequences. At least two non-identical *nos* gene clusters are found on the 26 genomes of *Schinkia* species, and *nos* gene clusters of representative strains (i.e., *Schinkia azotoformans* strain LMG 9581, and *Schinkia oryziterrae* strain ZYK) are shown.

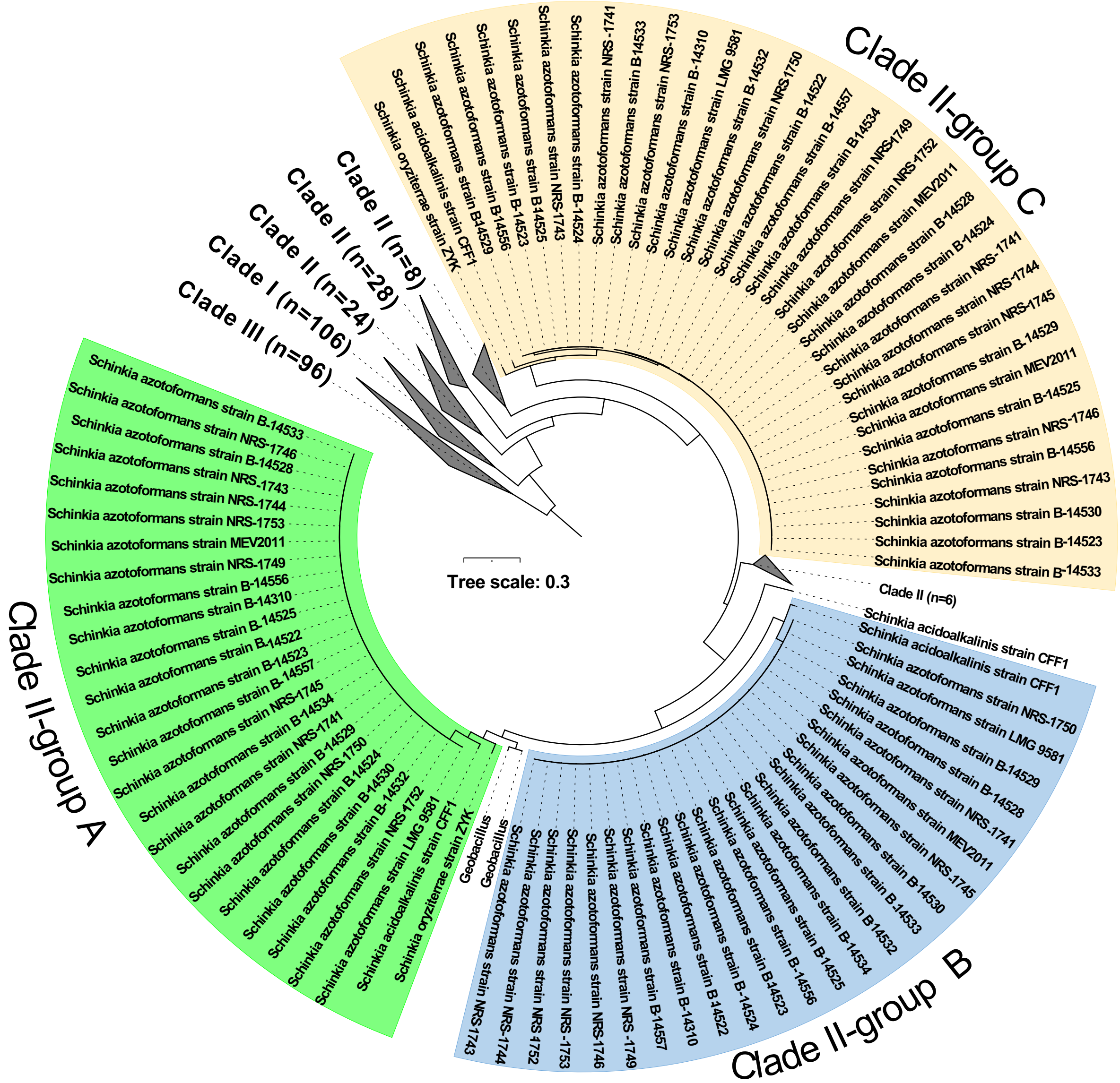

Fig. S6. Maximum likelihood phylogenetic tree based on 356 *nosZ* genes (105 Clade I, 68 clade II, and 96 clade III). The subclades comprising *Schinkia nosZ* genes are indicated in green (clade II-group A, 26 *nosZ* genes), blue (clade II-group B, 24 *nosZ* genes) and orange (clade II-group C, 35 *nosZ* genes). The tree scale bar represents the mean number of amino acid substitution per site. Select branches are collapsed for visual clarity.

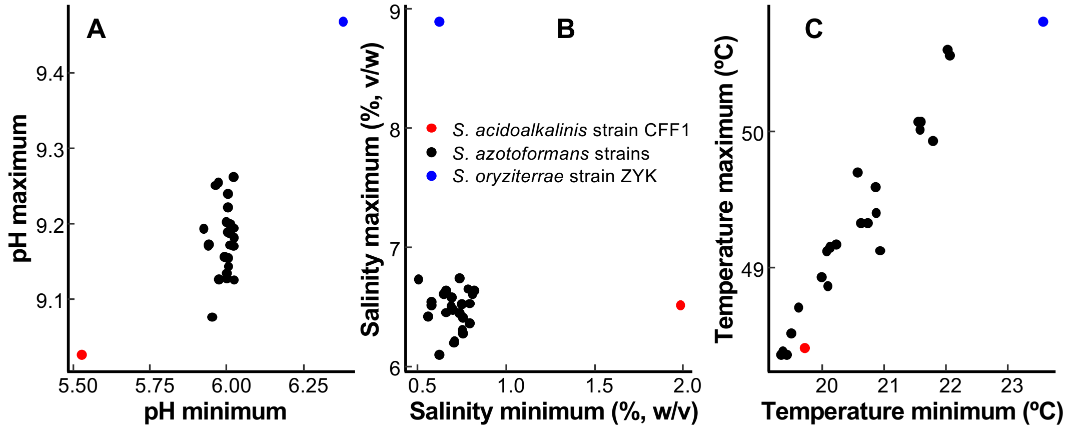

Fig. S7. Predicted growth requirements of *Schinkia* isolates based on amino acid composition of proteins encoded on the 26 available *Schinkia* genomes. Panels **A**, **B** and **C** show the minimum and maximum pH, salinity, and temperature predictions, respectively.

Supplemental References

1. Green AA. 1933. The preparation of acetate and phosphate buffer solutions of known pH and ionic strength, vol 55, p 2331-2336.
2. Kurtzer GM, Sochat V, Bauer MW. 2017. Singularity: Scientific containers for mobility of compute. PLOS ONE 12:e0177459. https://doi.org/10.1371/journal.pone.0177459
3. Martin M. 2011. Cutadapt removes adapter sequences from high-throughput sequencing reads. EMBnet J 17:3. https://doi.org/10.14806/ej.17.1.200
4. Callahan BJ, McMurdie PJ, Rosen MJ, Han AW, Johnson AJA, Holmes SP. 2016. DADA2: High-resolution sample inference from Illumina amplicon data. Nat Methods 13:581-583. https://doi.org/10.1038/nmeth.3869
5. Quast C, Pruesse E, Yilmaz P, Gerken J, Schweer T, Yarza P, Peplies J, Glöckner FO. 2012. The SILVA ribosomal RNA gene database project: improved data processing and web-based tools. Nucleic Acids Res 41:D590-D596. https://doi.org/10.1093/nar/gks1219
6. Bolyen E, Rideout JR, Dillon MR, Bokulich NA, Abnet CC, Al-Ghalith GA, Alexander H, Alm EJ, Arumugam M, Asnicar F, Bai Y, Bisanz JE, Bittinger K, Brejnrod A, Brislawn CJ, Brown CT, Callahan BJ, Caraballo-Rodríguez AM, Chase J, Cope EK, Da Silva R, Diener C, Dorrestein PC, Douglas GM, Durall DM, Duvallet C, Edwardson CF, Ernst M, Estaki M, Fouquier J, Gauglitz JM, Gibbons SM, Gibson DL, Gonzalez A, Gorlick K, Guo J, Hillmann B, Holmes S, Holste H, Huttenhower C, Huttley GA, Janssen S, Jarmusch AK, Jiang L, Kaehler BD, Kang KB, Keefe CR, Keim P, Kelley ST, Knights D, et al. 2019. Reproducible, interactive, scalable and extensible microbiome data science using QIIME 2. Nat Biotechnol 37:852-857. https://doi.org/10.1038/s41587-019-0209-9
7. Krakau S, Straub D, Gourlé H, Gabernet G, Nahnsen S. 2022. nf-core/mag: a best-practice pipeline for metagenome hybrid assembly and binning. NAR Genom Bioinform 4:lqac007. https://doi.org/10.1093/nargab/lqac007

8. Li D, Liu C-M, Luo R, Sadakane K, Lam T-W. 2015. MEGAHIT: an ultra-fast single-node solution for large and complex metagenomics assembly via succinct *de Bruijn* graph. Bioinformatics 31:1674-1676. https://doi.org/10.1093/bioinformatics/btv033
9. Chaumeil P-A, Mussig AJ, Hugenholtz P, Parks DH. 2019. GTDB-Tk: a toolkit to classify genomes with the Genome Taxonomy Database. Bioinformatics 36:1925-1927. https://doi.org/10.1093/bioinformatics/btz848
10. Jain C, Rodriguez-R LM, Phillippy AM, Konstantinidis KT, Aluru S. 2018. High throughput ANI analysis of 90K prokaryotic genomes reveals clear species boundaries. Nat Commun 9:5114. https://doi.org/10.1038/s41467-018-07641-9
11. Meier-Kolthoff JP, Carbasse JS, Peinado-Olarte RL, Göker M. 2021. TYGS and LPSN: a database tandem for fast and reliable genome-based classification and nomenclature of prokaryotes. Nucleic Acids Res 50:D801-D807. https://doi.org/10.1093/nar/gkab902
12. Zhou T, Xu K, Zhao F, Liu W, Li L, Hua Z, Zhou X. 2023. itol.toolkit accelerates working with iTOL (Interactive Tree of Life) by an automated generation of annotation files. Bioinformatics 39. https://doi.org/10.1093/bioinformatics/btad339
13. Letunic I, Bork P. 2024. Interactive Tree of Life (iTOL) v6: recent updates to the phylogenetic tree display and annotation tool. Nucleic Acids Res 52:W78–W82. https://doi.org/10.1093/nar/gkae268
14. Barnum TP, Crits-Christoph A, Molla M, Carini P, Lee HH, Ostrov N. 2024. Predicting microbial growth conditions from amino acid composition. bioRxiv doi:10.1101/2024.03.22.586313. https://doi.org/10.1101/2024.03.22.586313
15. Cantalapiedra CP, Hernández-Plaza A, Letunic I, Bork P, Huerta-Cepas J. 2021. eggNOG-mapper v2: functional annotation, orthology assignments, and domain prediction at the metagenomic scale. Mol Biol Evol 38:5825-5829. https://doi.org/10.1093/molbev/msab293
16. Seemann T. 2014. Prokka: rapid prokaryotic genome annotation. Bioinformatics 30:2068-2069. https://doi.org/10.1093/bioinformatics/btu153
17. Gilchrist CLM, Chooi Y-H. 2021. clinker & clustermap.js: automatic generation of gene cluster comparison figures. Bioinformatics 37:2473-2475. https://doi.org/10.1093/bioinformatics/btab007
18. Grant JR, Enns E, Marinier E, Mandal A, Herman EK, Chen C-y, Graham M, Van Domselaar G, Stothard P. 2023. Proksee: in-depth characterization and visualization of bacterial genomes. Nucleic Acids Res 51:W484-W492. https://doi.org/10.1093/nar/gkad326
19. Batth TS, Tollenaere MX, Rüther P, Gonzalez-Franquesa A, Prabhakar BS, Bekker-Jensen S, Deshmukh AS, Olsen JV. 2019. Protein aggregation capture on microparticles enables multipurpose proteomics sample preparation. Mol Cell Proteom 18:1027-1035. https://doi.org/10.1074/mcp.TIR118.001270
20. Dorfer V, Pichler P, Stranzl T, Stadlmann J, Taus T, Winkler S, Mechtler K. 2014. MS Amanda, a universal identification algorithm optimized for high accuracy tandem mass spectra. J Proteome Res 13:3679-3684. https://doi.org/10.1021/pr500202e
